# Plasmon-Enhanced Raman Sensing with Metal–Insulator–Metal Metasurfaces

**DOI:** 10.1021/acsami.5c25818

**Published:** 2026-04-09

**Authors:** Sümeyra Vural Kaymaz, Fahd Khalid-Salako, Hasan Sarıgül, Beyza Nur Günaydın, Hasan Kurt, Meral Yüce

**Affiliations:** † Department of Molecular Biology, Genetics, and Bioengineering, Faculty of Engineering and Natural Sciences, Sabanci University, 34956 Istanbul, Türkiye; ‡ Department of Materials Science and Nanoengineering, Faculty of Engineering and Natural Sciences, Sabanci University, 34956 Istanbul, Türkiye; § SUNUM Nanotechnology Research and Application Centre, Sabanci University, Istanbul 34956, Türkiye; ∥ Department of Biomedical Engineering, School of Engineering and Natural Sciences, Istanbul Medipol University, Istanbul 34810, Türkiye; ⊥ Research Institute for Health Sciences and Technologies (SABITA), Istanbul Medipol University, Istanbul 34810, Türkiye; # Department of Bioengineering, 4615 Imperial College London, South Kensington Campus, London SW7 2AZ, U.K.

**Keywords:** plasmonic, metal−insulator−metal, honeycomb, bowtie, nanotriangle, Raman spectroscopy

## Abstract

Metal–insulator–metal (MIM) plasmonic metasurfaces provide a powerful platform for enhancing light-matter interactions; however, achieving simultaneous spectral tunability, fabrication reproducibility, and ultrasensitive detection remains a challenge. Here, we present the rational design, simulation, and lithographic fabrication of three distinct MIM metasurfaces (bowtie, honeycomb, and nanotriangle) optimized for plasmon-enhanced Raman spectroscopy (PERS). Finite-difference time domain (FDTD) simulations reveal localized surface plasmon resonances with electric field enhancement factors (EF) exceeding *|E|* ^*2*^ ∼ 1600, supported by experimental reflection spectra and the fidelity of nanofabrication. Raman sensing of molecular probes (*R6G*, *4-ATP*, *4-CTP*) demonstrates analytical enhancement factors reaching 10^7^ and detection limits as low as ∼10^–15^ M. This is made possible by the designed nanogap resonances, and the broadband localized surface plasmon resonance (LSPR) overlap with the excitation and scattering bands. Our findings establish the lithographically defined MIM metasurfaces as reliable, tunable, and ultrasensitive surface-enhanced Raman spectroscopy (SERS) platforms, making them suitable for next-generation portable chemical and biological sensing systems.

## Graphical abstract

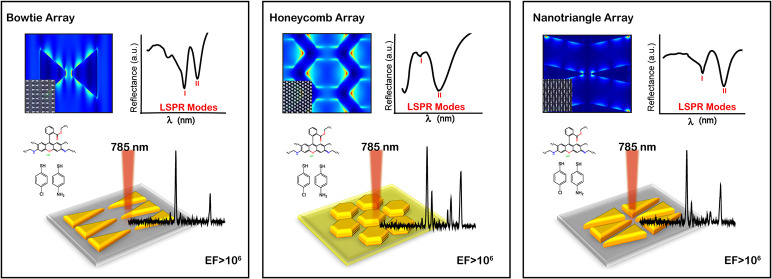

## 1 Introduction

Plasmon-Enhanced Raman Spectroscopy (PERS) addresses the poor efficiency of Raman scattering, providing exceptional sensitivity at the molecular scale. Raman spectroscopy yields distinctive spectral “fingerprints” of molecules derived from their vibrational energy states. However, this technique is fundamentally weak; the probability of Raman scattering often ranges from 10^–6^ to 10^–8^, making it frequently unfeasible to acquire significant signal in low-density samples. This constraint has been acknowledged as a significant impediment to analytical chemistry at low concentration limits and can be mitigated by inducing localized surface plasmon resonances on metallic nanostructures, resulting in pronounced electronic field intensification at specific resonance wavelengths.^1^ These plasmons, confined to the surface regions around the nanostructures, generate highly intense local electric field areas known as “hotspots” where Raman scattering is enhanced through electromagnetic, as well as chemical interactions, including charge transfer phenomena.^2^

Surface-Enhanced Raman Spectroscopy (SERS), referring to Raman sensing that is enhanced through the use of nanostructured metal surfaces, represents the most recognized and extensively utilized application of PERS, with practical applications in laboratory and industrial contexts.^3^ SERS enhances the Raman signal by increasing the local electromagnetic field strength around the analyzed substance, creating highly localized “hot spots,” that achieve Raman signal amplification up to 10^6^–10^12^ orders of magnitude, all while maintaining spectral selectivity and chemical fingerprinting. In the present study, engineered metasurface architectures are designed to exploit plasmonic coupling phenomena characteristic of PERS, and their experimental implementation corresponds to surface-based Raman measurements. The term “SERS” is therefore used throughout the manuscript to denote the analytical application.

Conventional plasmonic structures based on strong dipole–dipole interactions may not always be sufficient to provide significant near-field enhancement. The intensity of the scattered light is governed by both strong and weak coupling effects. Dark plasmon modes, such as quadrupole modes, have high resonance quality factors and can minimize light scattering. However, due to their weak interaction with scattered light, these modes are difficult to excite and detect in simple metallic nanostructures.^4^ To overcome these difficulties, plasmonic metasurfaces, consisting of periodically arranged subwavelength structures, have been developed. By optimizing the design and configuration of meta-atoms and integrating functional materials,^5,6^ these structures enable selective modulation over a wide range of the electromagnetic spectrum. Due to their ability to confine light at subwavelength scales and produce strong local field enhancements, plasmonic metasurfaces exhibit extraordinary sensitivity in detecting target molecules at nanometric and subnanometric levels.^7,8^ These nanostructures generate strong local electromagnetic fields, thereby creating areas commonly referred to as “SERS hot spots”.^9,10^ To further improve light interactions and increase the excitation of dark modes, metal–insulator–metal (MIM) nanostructures have attracted great attention.^11^ MIM configurations produce resonance spectra characterized by a Fano trough, resulting from the interference between bright and dark plasmon modes, which effectively excites dark modes. Known for their optical antenna properties, MIM structures minimize light scattering losses and enhance near-field effects, making them highly suitable for SERS applications.^12^

The practical deployment of MIM-based SERS substrates remains constrained by several well-documented limitations, including fabrication complexity, limited scalability, sensitivity of resonance conditions to nanometer-scale deviations, and poor interbatch reproducibility of hotspot distributions, limiting their real-world sensing applications.^13^ Additionally, the narrow resonance bandwidths of Fano-resonant MIM structures and their strong dependence on precise geometric alignment impose stringent fabrication tolerances that hinder iterative optimization and large-scale manufacturing.^14^ This work aims to address these challenges by establishing a systematic design-to-deployment workflow for MIM metasurfaces, integrating simulation-guided geometry design of simple, repeating arrays (entrenching nanoplasmonic hybridization and fairly broadband electric field enhancements); reproducible electron-beam lithography (EBL); and experimentally validated performance benchmarking across multiple antenna architectures.

Therefore, this study reports the simulation-backed design and fabrication, by EBL, of plasmonic MIM (aluminum-SiO_2_/Si_3_N_4_–Au) metasurfaces with three different geometric structures—Bow Tie Antenna (BTA), Honeycomb Array (HCA), and Nano Triangle Array (NTA). The central aim is a controlled tuning of gap resonances, scalable fabrication, and iterative optimization toward robust, application-ready SERS substrates suitable for practical chemical and trace-contaminant sensing. Accordingly, the SERS performances of metasurfaces were tested with well-known Raman probe molecules; 4-aminothiophenol (*ATP*), Rhodamine 6G (*R6G*), and 4-chlorothiophenol (*CTP*).

## 2 Materials and Method

A comprehensive experimental section is provided in the Supporting Information file (Section S1), detailing the simulation studies, fabrication procedures, optical characterization experiments, SERS measurement parameters, and the data processing pipeline.

## 3 Results and Discussion

### 3.1 Design, Parametric Optimization, and Analysis of MIM Metasurfaces

This study involved the design, simulation, and fabrication of plasmonic MIM substrates with various parameters, including Au antenna arrays on a film separated by a dielectric spacer, featuring multiple configurations and geometries. Numerous studies have shown that the MIM structure provides excellent performance in SERS because its resonance positions align with typical Raman excitation and scattering wavelengths, leading to significantly enhanced Raman scattering. The amplification mechanism of Raman signals can be understood by examining the distribution of the local electric field in plasmonic metasurfaces, constituting SERS “hotspots.”^12^ Therefore, the local electric field distributions within the designed structures were studied using full-wave simulations with the FDTD method (Ansys Lumerical FDTD, 2023 R1, Lumerical Inc.).

#### 3.1.1 Bowtie MIM Metasurfaces

A schematic illustration is presented in Figure 1, of the bowtie nanoarray integrated into a MIM stack consisting of a 75 nm SiO_2_ dielectric layer, a 100 nm Al layer, and a Ti adhesion layer. The 3D architecture emphasizes the key structural parameters of the bowtie elements. Figure 1A shows the final reflection spectrum over the range of 500 to 1200 nm, including the excitation laser wavelength of 785 nm used in the SERS analysis. SERS measurements were recorded over a 400–1800 cm^–1^ spectral window, corresponding to ∼810.45 nm–914.17 nm.

**Figure 1:**
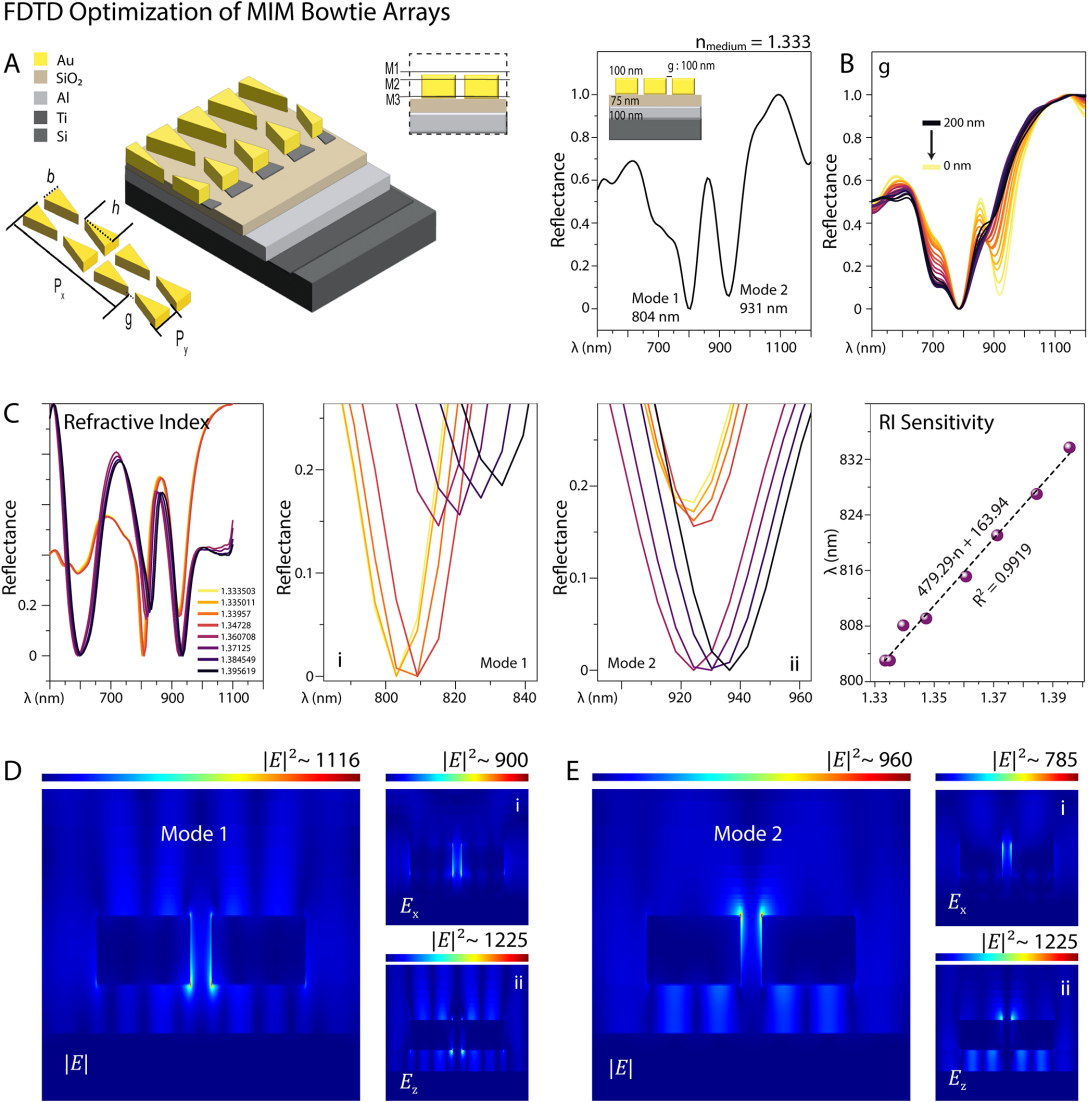
Geometrical tuning of MIM BTAs for dual mode plasmonic resonance. (A) Schematic illustration of the bowtie nanoarray integrated into a MIM architecture, highlighting key geometric parameters. (B) Parametric sweep of reflectance spectra as a function of nanogap size (g). (C) Refractive index-induced optical responses of the bowtie nanoarray, including (i) and (ii) reflectance dip shifts in modes 1 and 2 respectively; (iii) corresponding calibration curves derived from wavelength (mode 1) as a function of refractive index. (D–E). Electric field distribution maps at resonance wavelengths, showing |*E*|, *E*_*x*_, and *E*_*z*_ components in the *x*–*z* plane, highlighting dipolar field confinement and gap-enhanced localization.

Dielectric spacers play important roles in MIM nanoplasmonic substrates. Strong electric field enhancements occur from the coupling of localized plasmons on the nanostructures in the top layer, and their mirror images within the dielectric spacer.^15,16^ Similarly, localized plasmons and propagating polaritons on the back-reflector surface couple within the insulating layer, while the polaritons also initiate excitations in the nanoplasmonic hotspots, overall contributing to a greatly enhanced local field within the dielectric spacer.^15^ Accordingly, the back reflector and dielectric spacer materials and thickness in the designed substrates were modeled after an earlier reported MIM architecture in which the back reflector-dielectric spacer layers provided lossy waveguide-like optical properties, resulting in enhanced, hotspot-localized electric fields at wavelengths ∼800–850 nm.^11^ Additionally, FDTD simulations were conducted at varied spacer layer thickness (Figures S9 and S10) to optimize the nanoplasmonic response of the MIM architecture under the predetermined illumination parameters.

A substantial electric field enhancement (|*E*|^2^ ≈ 1400) was achieved at resonance wavelengths around 800 nm (LSPR Mode 1) and 920 nm (LSPR Mode 2) in a water environment (*n* = 1.33). Electric field analysis revealed strong dipolar behavior, particularly in the *E_x_* component within the nanogap region, indicating pronounced gap-mode coupling. Additionally, dipole resonances were observed at both the metal–medium and metal–dielectric interfaces. Field distributions examined in the *xy*-plane, just above (M1) and below (M2) the bowtie structures, confirmed distinct dipolar resonances in both the *x*- and *z*-components of the electric field (Figure S1E–F).

Parametric analysis showed that increasing the nanogap does not significantly affect the resonance near 800 nm, while it leads to the emergence of a second mode at longer wavelengths. Among geometric parameters, triangle height induces a strong red shift and enhances intensity in both modes, whereas base length has only a minor effect (Figure S1A–B). Periodicity also plays a critical role; increasing the horizontal periodicity (*P*_*x*_) results in a red shift, accompanied by a decrease in Mode 1 intensity and an increase in Mode 2. In contrast, varying the vertical periodicity (*P*_γ_) produces the opposite trend (Figure S1C–D).

Upon examining the dielectric layer, we discovered distinct electric field propagation characteristics along all three axes. EF of a SERS substrate is defined as |*E*_loc(excitation)_|/|*E*_0_|^2^ × |*E*_loc(stokes)_|/|*E*_0_|^2^, representing the product of the near-field enhancement at the excitation and Stokes wavelengths.^17^ Therefore, the presence of a broadband LSPR envelope that overlaps with both excitation (785 nm) and Stokes-shifted emission is critical for high-performance SERS. The close spectral proximity of Mode 1 to the excitation wavelength, along with broadband enhancement covering the Raman region, confirms the suitability of this MIM bowtie metasurface for achieving strong dual-mode electric field amplification.

#### 3.1.2 Honeycomb MIM Metasurfaces

Figure 2 represents the design and optimization of the MIM HCA, focusing on how key geometrical parameters affect optical response and field enhancement. The simulated structure (top left) consists of Au hexagonal elements arranged in a honeycomb configuration on top of a multilayer stack of Si_3_N_4_ (150 nm), Al (100 nm), Ti, and a silicon wafer. The hexagons feature slightly rounded corners to more accurately reflect the actual geometries of fabricated objects.

**Figure 2:**
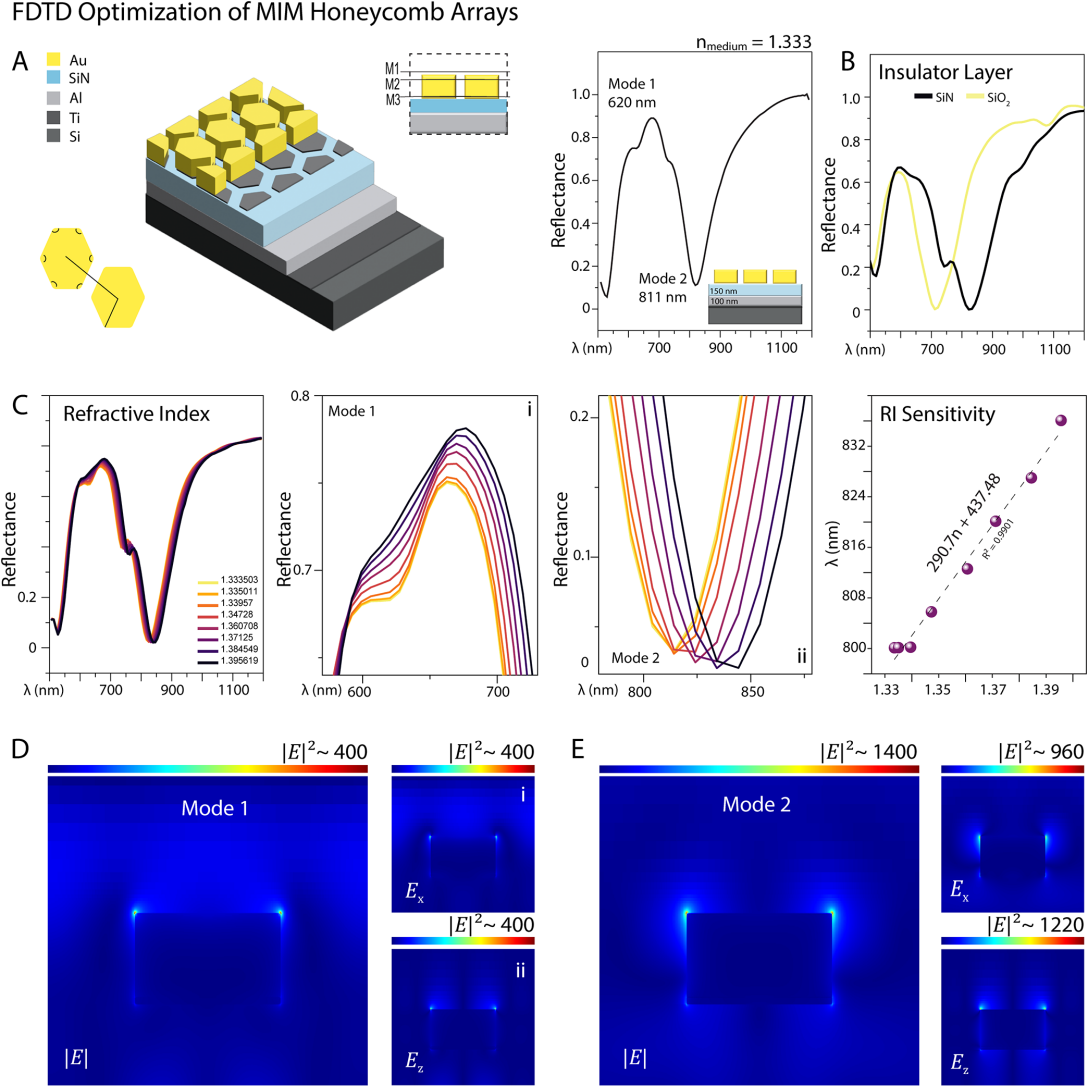
Design, optimization, and simulation results for the HCA on the MIM layer. (A) Schematic of the hexagonal gold nanoarray embedded in a multilayer metal–insulator–metal (MIM) architecture, consisting of a Si_3_N_4_ dielectric spacer, Al back-reflector, Ti adhesion layer, and silicon substrate. (B) Influence of dielectric material on the optical response. Replacing SiO_2_ with Si_3_N_4_ enhances Mode 2, leading to stronger field confinement and increased resonance intensity. (C). Refractive index-induced optical responses, including (i) and (ii) reflectance dip shifts in modes 1 and 2 respectively; (iii) corresponding calibration curves derived from wavelength shifts (mode 2). (D–E). Electric field distributions in the *x*–*z* plane at resonance wavelengths, showing *|E|, E_x_*, and *E*_*z*_ components, highlighting localized field enhancement and interface-confined plasmonic behavior.

Two LSPR modes are identified in the reflectance spectrum (Figure 2A): Mode one at ∼620 nm and Mode two near ∼800 nm. These modes arise from the geometry-dependent coupling of incident light to plasmonic modes at the metal–dielectric interface. As the hexagonal diameter (D) increases, both resonances redshift (Figure S2 C), consistent with an increase in the effective optical path length.

The corner radius (*r*) varied from 0 to 60 nm (Figure S2 A) to evaluate its impact on fabrication fidelity and optical performance. While increasing r makes the hexagons more circular and easier to fabricate via EBL, it was found to have a negligible influence on resonance positions. A value of 20 nm for radius was thus selected as a practical compromise between performance and manufacturability.

Variation in the Au thickness (Figure S2B) results in a redshift and broadening of the plasmonic resonances, with an optimal response observed around 120 nm. As shown in Figure 2B, replacing the dielectric spacer layer with Si_3_N_4_ (*n* ≈ 2.0) instead of SiO_2_ (*n* ≈ 1.46) and tuning its thickness significantly enhances field confinement at the metal–dielectric interface (Figure S2D). HCA-MIM exhibits a limited dispersive sensitivity to refractive index variations, responding primarily through changes in resonance amplitudes rather than wavelength shifts, thereby providing a stable and predictable optical response. (Figure 2C).

Electric field simulations reveal strong field localization in the nanogaps between adjacent hexagons, particularly for Mode two. Three *XY* field-monitoring planes (*M*_1_, *M*_2_, and *M*_3_) were positioned above the gold surface, within the gold, and at the dielectric interface, respectively. Field maps of *|E|*, *E_x_*, and *E*_*z*_ in both the *xz*- and *xy*-planes indicate distinct spatial confinement (Figure 2D,E). Mode one shows strong localization near the tips of the hexagons, while Mode two displays a broader distribution across the array, with peak *|E|* ^*2*^ values approaching 1400 (Figure 2E). The spectral behavior of SERS surfaces was investigated by the FDTD method, and resonance wavelengths and Cartesian electric field distributions were reported. Since the frequency of Raman scattering is very close to the frequency of the incident light, it is also frequently defined as *|E|* ^4^ in the literature.^18^ The highest EF ratio is obtained when the frequency of the incident light matches the LSPR frequency. These results demonstrate that the honeycomb metasurface supports broadband and spatially uniform field enhancement, with resonance alignment near the Raman scattering region. This design enables efficient excitation and emission enhancement in SERS, leveraging the fourth-power dependence of electric field intensity on signal amplification.

#### 3.1.3 Nanotriangle MIM Metasurfaces

The primary distinction between the bowtie and nanotriangle models is in the configuration of the isosceles triangles. The geometric parameters of the nanotriangle were maintained consistent with the bowtie model; however, periodicities in the x and y planes (Figure 3) were optimized. The schematic shown in Figure 3A illustrates an NTA integrated into the MIM structure. The nanotriangle elements are arranged in a back-to-back configuration, which enhances the near field coupling between them. In Figure 3B–C, parametric sweeps demonstrate how changing the periodicity in the *x*-direction and the *y*-direction affects the resonance wavelengths. As the spacing between elements increases, the resonance wavelengths shift toward longer wavelengths, a result of weaker near-field coupling and a greater optical path length.

**Figure 3:**
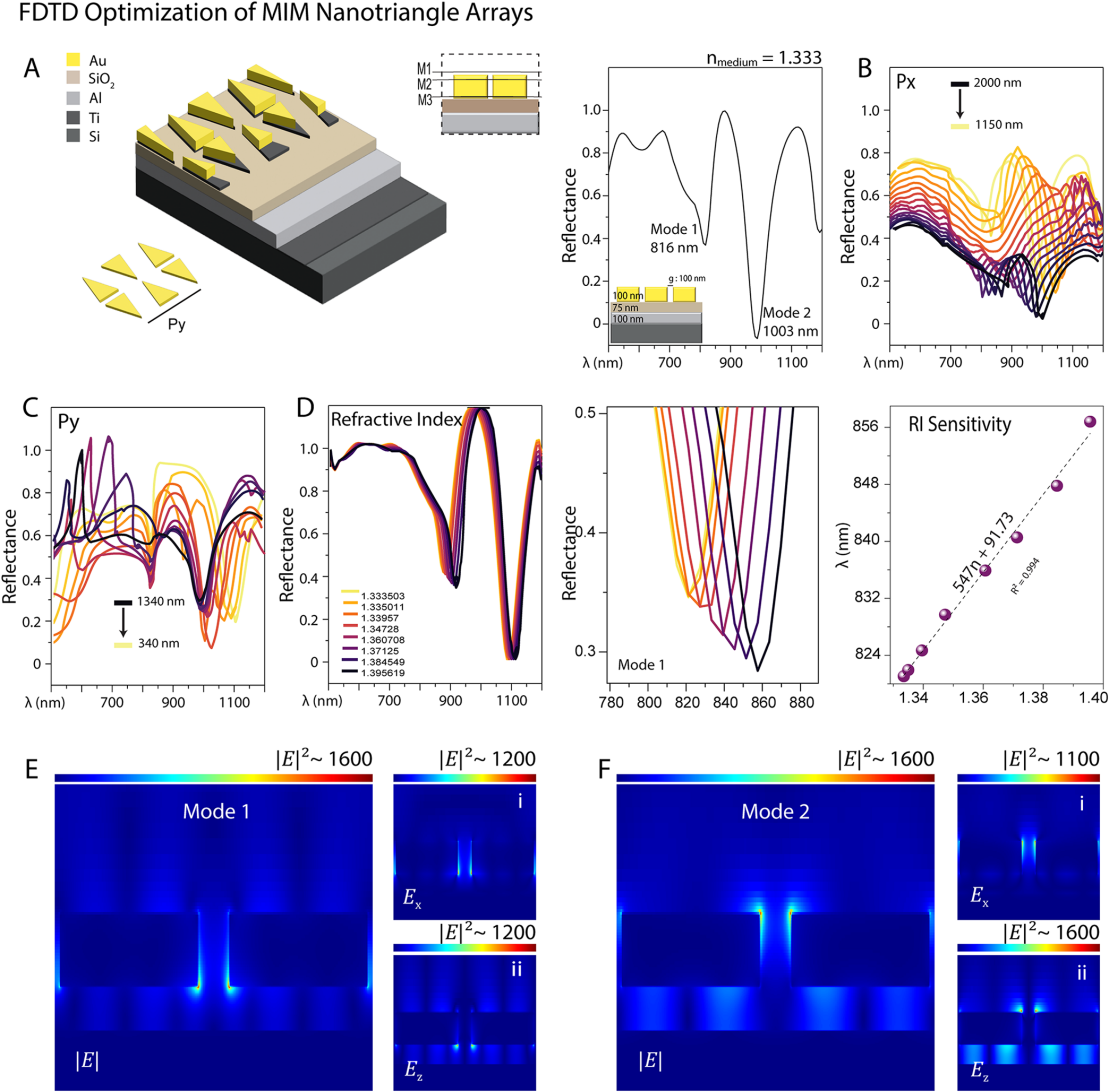
Design, optimization, and simulation results for the NTA on the MIM layer. (A). Schematic of the nanotriangle array on a multilayer MIM architecture and the back-to-back nanotriangle configuration promotes strong near-field coupling. The inset shows a cross-sectional view of the multilayer configuration. (B–C). Parametric sweeps illustrating the influence of periodicity along the *x*-direction (*P_x_*) and *y*-direction (*P*_γ_). (D). Refractive index-induced optical responses, including an enlarged panel showing the reflectance dip shifts in mode 1 specifically and a corresponding calibration curve of reflectance dip peak against refractive index. (E–F). Electric field distributions in the *x*–*z* plane at resonance wavelengths, showing |*E*|, *E_x_*, and *E*_*z*_ components, highlighting dipolar field confinement and interface-localized enhancement.

The electric field distributions in the xz-plane for both resonance modes are shown, including the |*E*|, *E_x_*, and *E_z_* components Figure 3E-F. Field maps in the *xy*-plane are extracted from three monitor planes -*M*_1_ (above the Au), *M*_2_ (within the Au), and *M*_3_ (between Au and dielectric)- revealing the spatially resolved modal profiles. Mode one exhibits strong field confinement within the nanogaps, while Mode two shows a broader, more hybridized distribution Figure S1G,H.

LSPR responses of the nanotriangle, bowtie, and honeycomb metasurfaces each offer unique advantages for SERS. In the NTA, the back-to-back configuration of isosceles triangles promotes enhanced near-field coupling in the nanogaps, resulting in broad dual-mode resonances that significantly amplify the electromagnetic field upon excitation. In contrast, the bowtie design exhibits strong dipolar confinement at the tips, providing broadband enhancement that efficiently overlaps with both the excitation and Raman scattering wavelengths. Meanwhile, the HCA leverages its hexagonal symmetry to generate a dense network of plasmonic hotspots, ensuring high signal ensemble reproducibility across the sensing surface. Collectively, these complementary architectures demonstrate that careful geometric tuning of MIM metasurfaces can be exploited to achieve both strong and broadband electromagnetic enhancement, thereby enabling trace-level molecular detection in SERS applications.

### 3.2 Fabrication and Characterization of Plasmonic Metasurfaces

MIM substrates in nanoplasmonic substrates have previously been explored in colloidal nanoparticle core–shell and nanoparticle on a mirror designs,^19,20^ exploiting the coupling of dipoles around metal nanoparticles and their images from a back-reflector metal.^16^ While these designs achieved simple MIM-like architectures with sufficient nanoplasmonic enhancement of Raman scattering, practical SERS applications require substrates that can be rapidly fabricated, at scale, using simple yet effective design parameters that ensure uniform hotspot density and distribution; an outcome not reliably achieved with colloidal substrates. These factors are essential for translating SERS substrate research into real-world, field-deployable sensing platforms, particularly for trace-contaminant and chemical detection in dynamic environments. Therefore, we adopted an EBL based workflow for the fabrication of the MIM substrates, while optimizing the design parameters of the metasurfaces accordingly. Given our intention to develop these substrates ultimately for field deployment, the EBL approach ensures that the substrates can be fabricated reproducibly, with high batch sizes on large wafers, while maintaining consistent plasmonic hotspot distribution.

The top portion of Figure 4A presents a schematic of the sequential fabrication steps. The MIM layers were deposited on silicon substrates and subsequently patterned via EBL followed by a lift-off process to produce bowtie, honeycomb, and nanotriangles. Figure 4C–E displays a combination of simulation and experimental data. FDTD simulations provide electromagnetic field distributions, while SEM images confirm the fidelity of the fabricated structures. Reflection spectra were recorded using a custom-built LSPR-based optical setup (Figure 4B) that integrates a CMOS camera, coupled with an achromatic lens in the imaging arm, and an apochromatic tube lens with a cooled Andor camera in the spectroscopy arm. A specialized spectrometer operating in the 780–850 nm range facilitated the sensitive detection of the reflection dips.^11^

**Figure 4:**
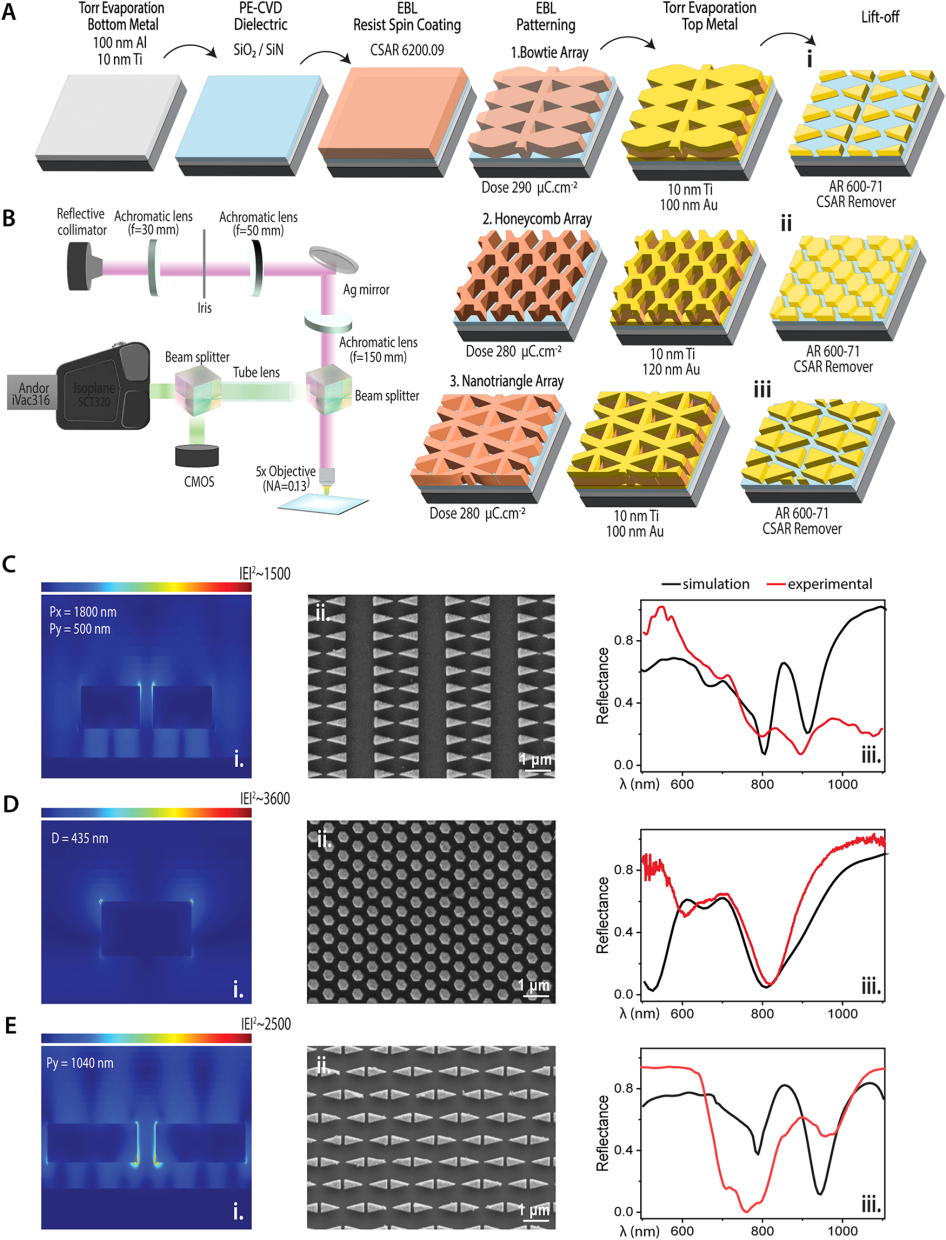
Fabrication and optical characterization of MIM metasurfaces. (A) Schematic of the lift-off process used to create bowtie, honeycomb, and nanotriangle metasurfaces. (B) Diagram of the custom-built reflectance measurement setup. (C–E). Secondary electron images and reflection spectra for the BTA (C); HCA (D); and NTA (E).

For BTA (Figure 4C), the simulated electric field distribution reveals intense plasmonic hotspots at periodicities of *P_x_* = 1800 nm and *P*_γ_ = 500 nm. In the case of HCA (Figure 4), pronounced near-field intensification is observed along the boundaries of the hexagonal elements (*D* = 435 nm). NTA (Figure 4E) exhibits a distinct field distribution with localized hotspots in specific regions (*D* = 1040 nm).

While the experimental reflection spectra exhibit an overall good agreement with the simulation results, a slight blue shift is evident in the measured data. This discrepancy is attributable to several factors: (i) Fabrication Imperfections: Variability in actual nanostructure dimensions, edge rounding, and surface roughness may lead to deviations from the idealized geometries assumed in the simulations. (ii) Material Property Variations: Differences between the optical constants of the deposited films and those reported in the literature (e.g., Palik data) can alter the effective refractive index, thereby shifting the resonance wavelengths. (iii) Process Variability: Inconsistencies in layer thicknesses and the precision of the lift-off process can modify the effective optical path lengths, resulting in resonance shifts. Collectively, these factors account for the observed discrepancies and highlight the inherent challenges in achieving exact congruence between simulation and experimental data. Nevertheless, the strong overall correlation validates the design and fabrication strategy, underscoring the potential of these metasurfaces for highly sensitive Raman detection.^21–23^

The optical measurements, in accordance with FDTD findings, validate the reproducible fabrication of MIM metasurfaces with demonstrable LSPR modes as presented in Figure 4. The plasmonic behavior of the metasurfaces was further characterized by studying their LSPR refractive index sensitivity, using glycerol–water solutions (Figure S3). Various glycerol–water solutions from 1% to 50% were formulated, and the refractive index of each solution was determined using a refractometer, resulting in values from 1.3330 to 1.3956 (Table S2). Reflectance spectra obtained under p-polarized illumination exhibited two predominant resonant modes (Mode one and Mode two) across all designs. In all three substrates, Mode one demonstrated redshift responsive to variations in the ambient refractive index. The refractive index response of Mode two varies according to the geometry. Specifically, in the BTA, Mode two exhibited a significant dispersive shift, similar to Mode one, indicating the robust coupling of this design to its surrounding environment. No notable wavelength shift was detected in Mode two within the Honeycomb and NTAs; the response was confined to variations in the depth and intensity of the reflection dips. Consequently, although Mode one ensures consistent dispersive sensitivity across all designs, the characteristics of Mode two fluctuate according to the geometric configuration; bowtie structures demonstrate elevated refractive index sensitivity on surfaces, whereas honeycomb and nanotriangle designs display a predominantly amplitude-based response.

The discrepancies observed between the simulated and experimental reflectance spectra can be attributed mainly to the optical collection conditions inherent in the optical measurement system. In the simulations, a periodic nanostructure array measuring 60 μm × 60 μm was analyzed, under the assumption of ideal plane-wave excitation and signal collection exclusively from the patterned region. The experimental measurements indicated that the Andor iVac camera and microscope objective captured light from both the nanostructured array and the neighboring unpatterned flat surface. This results in two significant implications: The initial contribution pertains to the background. The flat surface fails to support the lattice resonances of the nanostructured array. It generates a broadband reflection response that contributes to the signal from the nanostructure. This diminishes the contrast of the resonance dips, resulting in a spectrum that appears broader than that observed in the simulation. Additionally, finite size and illumination effects are present. The illuminated spot size, as determined by the laser beam waist width and the objective numerical aperture, exceeds the dimensions of the array. Consequently, a portion of the excitation engages in the unpatterned areas. Furthermore, alignment errors and surface roughness caused by fabrication contribute to increased scattering and a broader resonance profile. The experimentally observed resonance is consequently weaker and spans a wider spectral range compared to the idealized simulation.

### 3.3 SERS Performance Evaluation of Plasmonic Metasurfaces with Probe Molecules

#### 3.3.1 SERS Enhancement Factor

SERS signal depends on the laser power, excitation time, and number of molecules excited. Since direct comparison at different excitation wavelengths can lead to errors, SERS and reference Raman measurements for the same wavelength were performed with fixed parameters. This way, EF can be used to evaluate SERS performance in different experimental conditions. A well-accepted and widely used definition is the ratio of the SERS intensity (*I*_SERS_) of a molecule to the normal Raman intensity (*I*_REF_) of the same molecule in the absence of the substrate, with the following formula^24^

$$\mathrm{EF}=\left(I_{\mathrm{SERS}}/N_{\mathrm{SERS}}\right)/\left(I_{\mathrm{REF}}/N_{\mathrm{NORM}}\right)$$

Where *N*_SERS_ and *N*_NORM_ are the number of molecules detected in the SERS and normal Raman measurements of SERS and reference samples at 1090 cm^–1^ for the 4-ATP molecule, for all measurements, an ×60 objective with a numerical aperture of 0.85 was employed to conduct Raman measurements. The EF obtained based on 4-ATP measurements on BTA, HCA, and NTA substrates was calculated to be ∼10^6^ (Supporting Information – S2). At increased laser power, the observed signal may appear to be saturated as a result of the diversion of energy that would typically contribute to the Stokes Raman signal to Anti-Stokes signals. Analyte degradation, which reduces enhancement, may occur with further power increases. The calculations show that the EF value is 10^5^–10^6^ based on the intensity values obtained from the samples with a concentration of 10^–11^ M, under conditions where the laser power falling on the sample is only 5 μW. This result reveals that the metasurfaces offer a very high performance in enhancing the Raman signal, with significant potential for ultralow concentration SERS sensing. The structural features of the fabricated metasurfaces significantly contribute to the increase in the Raman signal by promoting strong near-field interactions and the formation of hotspots. Additionally, the experimental/calculated EFs are within the same order of magnitude as the simulated/theoretical EF values, implying the fidelity of the fabrication process and the realization of rationally designed nanoplasmonic SERS substrates (Table S1).

These metasurfaces offer significant advantages in terms of SERS and optical sensor applications. Compared to similar studies, achieving an EF value of 10^6^ demonstrates that this method provides both a powerful and practical solution for nanoplasmonic biosensing applications.^25–28^ Although studies with higher EF values exist, they often have disadvantages, including fabrication complexity, repeatability issues, and stability concerns. Wang et al., in their research using ordered silver nano cube (NC) superlattice metasurfaces, enable wearable sensor design thanks to flexible polymer and hydrogel-based support structure. However, since the fabrication process of this structure requires the formation of a superlattice of self-organized nano cubes, its repeatability and control are challenging.^29^ Chen et al. introduced the use of a porcelain carbon nanowire array (PCNA) as a new substrate for SERS. This surface is compatible with biomolecules due to its carbon-based structure and is characterized by its ability to quench fluorescence. However, the PCNA structure generally exhibits low electric field enhancement, a narrow bandwidth, and a complex fabrication process, which can be limiting in some cases. This may cause difficulty in achieving very high signal enhancement levels, especially those obtained with conventional SERS.^30^

#### 3.3.2 Probe Molecule Detection Performance

To comprehensively evaluate the SERS performance of our plasmonic metasurfaces, we selected three probe molecules—*4-ATP*, *R6G*, and *4-CTP*, based on their distinct Raman fingerprints (*4-ATP* and *4-CTP* being thiophenol derivatives), varied adsorption behaviors, and relevance for chemical and biological sensing.^31–33^ The probe molecules utilized were carefully selected to assess the performance of metasurfaces across fairly diverse chemical and electronic circumstances. Importantly, R6G, a relatively large molecule, presents a distinct surface chemistry dominated by physisorption–desorption equilibrium, in contrast to 4-ATP and 4-CTP, both smaller molecules with thiol groups, capable of stable self-assembly on the gold layer.^34^ The dissimilarity in electronic character of the terminal substituents of 4-ATP (electron-donating NH_2_ group) and 4-CTP (electron-accepting Cl moiety) provides further diversification, enabling the validation of the sensing substrates’ performance across adsorption regimes, including physisorption and self-assembly; slightly reinforced or weakened by electron-donating and accepting terminal groups, respectively.^32^

SERS experimental setup and relative optimization, construction and calibration were previously published within our group, as briefly outlined in Section S1.4.^35^ Raman spectra were recorded over the range of 400–1800 cm^–1^ with a 1-s exposure and 30 accumulations per measurement. To ensure data reliability, at least 10 measurements were performed on different 60 μm × 60 μm regions of the nanoplasmonic array. Representative SERS spectra of *4-ATP* over concentrations ranging from 10^–15^ to 10^–7^ M on the three different metasurfaces are presented in Figure 5, alongside calibration curves obtained from Raman scattering intensity (peak height) at a characteristic shift (∼1090 cm^–1^), fitted against concentration with a logistic function.

**Figure 5:**
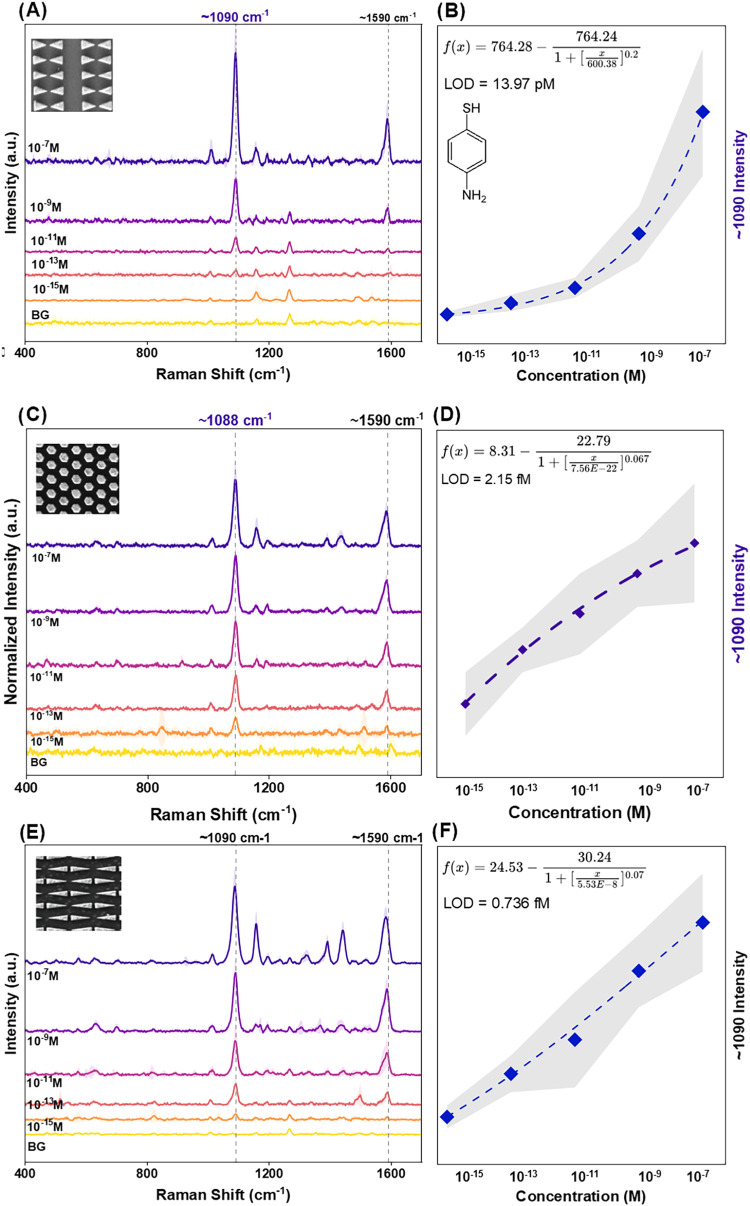
4-ATP SERS Sensing on the fabricated MIM metasurfaces. (A, C and E) Representative spectra obtained on the NTA, HCA, and BTA respectively. (B, D, and F) 4-ATP Calibration curves on the NTA, HCA, and BTA respectively.

The 4-ATP SERS spectra generally show clearly observable characteristic peaks at 1090 cm^–1^ and 1590 cm^–1^, both in-plane, in-phase Raman-active modes of 4-ATP (a_1_), attributable to Carbon–Sulfur (ν-CS) and aromatic ring (ν-CC) stretching, respectively.^33,36^ Notably, out-of-phase modes (b_2_) at ∼1160 cm^–1^ (δ-CH) and 1435 cm^–1^ (δ-CH + ν-CC) only begin to appear at higher 4-ATP concentrations, demonstrating the dominance of electromagnetic (EM) enhancement over charge-transfer mechanisms.^33^ These findings are consistent with the observed congruence of simulated and experimentally obtained EF values. The SERS substrates have been rationally designed and fabricated to leverage EM enhancement, such that the LSPR aligns with the Raman scattering band (Stokes shifts) and the excitation wavelength. Consequently, the plasmon resonances produced during laser excitation not only enhance the local field of the incident light but also provide an additional amplification mechanism through the concurrence of Raman-scattered waves with the resonance region—the double-resonant configuration results in a predominant electromagnetic impact and a significant enhancement of the resultant Raman signals. Thus, the robust SERS response demonstrated by the MIM metasurfaces is attributable to the LSPR-Raman overlap, which enhances the EM contribution.

The metasurfaces realized limits of 4-ATP detection in the femtomolar (NTA and HCA) and picomolar (BTA) ranges, demonstrating low concentration detection in the ideal case of small, aromatic, thiolated molecule sensing, with an electron-donating terminal group in the para (γ) position. Comparing calculated EF and LOD values across the three surfaces also demonstrated significant SERS-enhancement of 4-ATP sensing on the surfaces, with the EF values varying inversely with LOD (Figure 6).

**Figure 6:**
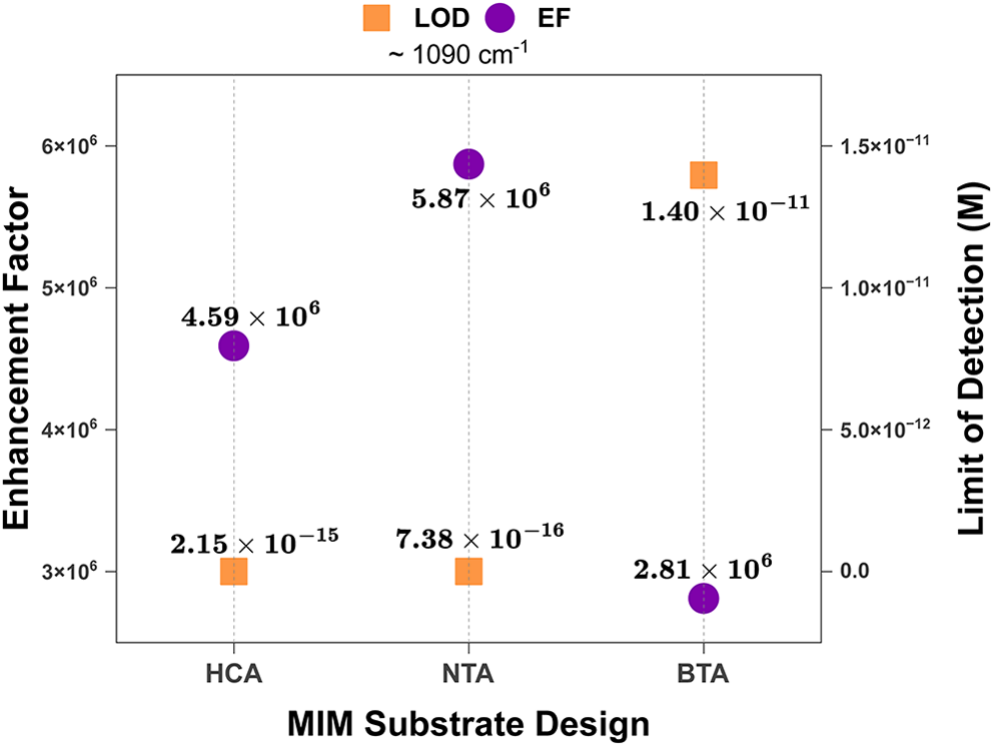
A simple comparison of EF and LOD values obtained from 4-ATP sensing on the three SERS substrates.

NTA, with the highest EF demonstrated subfemtomolar detection limit, while HCA and BTA, with lower EF values, also demonstrated lower sensitivity (fM and pM, respectively). This suggests a possible mechanistic link between EF and detection sensitivity, demonstrably enhanced primarily by EM mechanisms, especially at lower concentrations. However, it is worth noting that simple mathematical or quantitative derivations cannot be made to describe the relationship between EF and LOD within the scope of this study. While EF calculations essentially compare signal intensities, normalized against the number of illuminated probe molecules, LOD—the way we have computed it, is further dependent on signal variability across repeat measurements and noise levels represented by blank measurements, which are influenced by geometrical differences across the metasurfaces. Additionally, the three substrates report EF values in similar orders of magnitude (∼10^6^). Given that the discriminating value of EF in SERS performance assessment lies primarily in its order-of-magnitude regime, rather than minor numerical fluctuations, any perceived dissimilarities in the SERS enhancement on the various substrates are trivial and can neither satisfactorily explain the different detection limits achieved, nor statistically compare the SERS performances of each substrate relative to the others.

Notably, the geometrical parameters of the surfaces significantly alter their adsorption properties (Section S10). While the EF values obtained were derived under the assumption of substrate monolayer coverage, adsorption isotherm analyses demonstrated that around the LOD concentrations (pM–fM), fractional surface coverages (θ_LOD_) were (Figure S11)­

- 0.1 at 10 pM on BTA
- 0.1 at 1 fM on NTA
- ∼0.3 at 1 fM on HCA

Accordingly, a (1/θ_LOD_) correction factor is required to be applied to the calculated analytical EF, compensating for its underestimation at submonolayer coverage levels. Consequently, the fraction adsorption-corrected analytical EF at their respective LOD of the metasurface designs are

- HCA (at fM LOD): Corrected EF = 3.33 × 7.38 × 10^5^ = 2.46 × 10^6^
- NTA (at fM LOD): Corrected EF = 10 × 1.01 × 10^6^ = 1.01 × 10^7^
- BTA (at pM LOD): Corrected EF = 10 × 4.53 × 10^5^ = 4.53 × 10^6^

Similarly, we compared the 4-CTP sensing performance of the three substrates, at concentrations ranging from 10^–15^ to 10^–7^ M. Representative spectra of 4-CTP and calibration curves obtained from the three substrates are presented in Figure S12.

The 4-CTP response reveals vibrational modes at ∼536 (ν-CCl), 1074 (δ-CH), 1095 (ν-CS), 1110 (ν-CH), and 1568 cm^–1^(ν-C=C). The relatively low stability of CTP–Au pseudocovalent bonding reflects in the marked absence or weakness of these peaks at lower concentrations, up to 1 nM, especially on the NTA substrate. Interestingly, the HCA substrate, which geometrically presents a higher general surface area coverage by gold and more nano plasmonic hotspots per unit area compared to NTA and BTA, demonstrates better sensing performance of the chlorinated thiophenol, reporting picomolar sensitivity, while NTA demonstrates markedly reduced sensitivity at ∼1 nM. Finally, we compared the R6G sensing performance of the three substrates as presented in Figure S13.

R6G consists of a xanthene ring core conjugated with aromatic substituents and an amine-functionalized diethylamino group, providing multiple potential adsorption sites and pathways for plasmon–molecule coupling. Vibrational bands characteristic of R6G appear at ∼ 610, 770, and 1363 cm^–1^ on the NTA and BTA substrates, while HCA demonstrated enhancement of the slightly red-shifted in-plane bending mode (∼600 cm^–1^), as well as the in-plane xanthene ring breathing + δ­(NH) + ω­(CH) mode at ∼1309 cm^–1^ and the xanthene ring stretching, ν­(CN), δ­(CH) + δ­(NH) mode at ∼1499 cm^–1^,^37^ both of which were not observed on NTA and BTA. The ring deformations; coordinated xanthene ring breathing; C–N stretching; and mixed C–H/N–H deformation modes of R6G are sensitive to adsorption orientation and local field polarization. On the HCA substrate, the selective enhancement of the ∼1309 and ∼1499 cm^–1^ modes suggests a configuration in which the xanthene core is more strongly coupled to the local electromagnetic fields, favoring vibrations involving delocalized π–electron motion and amino-group involvement, potentially due to enhanced multipolar coupling and more distributed hotspot environments. In contrast, the dominant enhancement of lower-frequency ring deformation modes on NTA and BTA indicates preferential enhancement of modes associated with more localized aromatic distortions, consistent with stronger hotspot confinement and a more rigid molecular adsorption geometry. These results align with earlier SERS fingerprints of the R6G dye,^38,39^ and demonstrate the influence of molecular adsorption geometry, field localization scale, and hotspot topology on mode-selective enhancement in SERS sensing, leading to geometry-dependent spectral expression and sensitivity variations. Accordingly, the sensitivity of HCA was at the pM level for R6G, while NTA demonstrated higher (fM) sensitivity, and BTA showed the least sensitivity (∼200 nM).

Across the different substrates, high statistical variability was occasionally observed at low concentrations (most prominently at Figures S12F and S13D,F). These arise primarily from intrinsic statistical variability associated with SERS quantification at trace levels. At picomolar and femtomolar concentrations, molecule adsorption onto nanoplasmonic hotspots exhibit stochastic behaviors, leading to signal fluctuations, and manifesting as wider confidence intervals in regions approaching the noise floor. While this results in reduced precision in the extreme low-concentration tail, it is important to note that the calibration curves obtained from the logistic fits remain monotonic and statistically distinguishable from the blank, supporting validity of the reported detection thresholds. Therefore, LOD values should be interpreted primarily as reliable detection capability rather than absolute quantitative precision, consistent with accepted analytical conventions.^40^

To assess the practicality of SERS detection of each analyte on the fabricated substrates, an overlay of background spectra and analyte spectra at respective concentrations close to the calculated LOD values are presented in Figure S14, with the representative SERS peaks whose intensities constitute the calibration curves annotated. The apparent differences in SERS intensities at these peak positions for all analytes on the BTA and NTA substrates demonstrate the practical detection ability of the SERS substrates at concentrations close to their detection limits. However, care must be taken to interpret the detection limits of the HCA substrate, considering how especially noisy its background signals are. Although SERS intensities at the representative peak regions are higher than in the respective background spectra, high variances in adjacent background spectral regions impose the risk of false positives in blind sample measurements, at concentrations approaching the detection limit. Assignments of the peaks observed from the three probe molecules on the MIM substrates are provided in Table S4.

Notably, spectra obtained from the SERS measurements reveal peaks that cannot be attributed to the analytes, indicating the presence of artifacts. These may arise from residual resist that persists on the surface following the CSAR 62 lithography process. Residual materials that remain postdevelopment or lift-off processes could display intrinsic Raman-active characteristics owing to the presence of aromatic and halogenated functional groups. These residues can introduce supplementary vibrational modes in the spectra, potentially overlapping with the signals of the analyte. Raman spectra of residual resist potentially remaining on the surface were compared with literature data, confirming that some observed signals were attributed to background contributions (Figure S7). The effects of these artifacts cannot be eliminated; however, their impact on the results was minimized in our study through regular surface cleaning, controlled fabrication protocols, and systematic background subtraction methods. The SiO_2_ layers utilized in bowtie and nanotriangle designs, along with the Si_3_N_4_ substrates favored in HCAs, contribute to distinct vibrational modes in the spectra that are unrelated to the analyte, in addition to lift-off residues. This is a primary factor contributing to the observed differences in background signals among the three distinct designs. The intrinsic phonon vibrations of SiO_2_ and Si_3_N_4_ contribute weakly yet selectively to the Raman spectra, complicating peak identification, particularly in low-intensity analyte measurements. Thus, lithography-induced resist residues and the substrate materials may contribute to the presence of nonanalyte signals in the spectra. In this study, these effects were systematically evaluated as background contributions through a comparison with spectral data documented in the literature.

Across all three probe molecules, the HCA metasurface exhibits consistent femto-picomolar level sensitivity (10^–12^–10^–15^ M) due to pronounced localized plasmonic enhancements on a spatially homogeneous substrate, with well-distributed and abundant hotspots. Surface attachment artifacts arising from the chemical and electronic disparities in the probe molecules appeared to have a minimal effect on its sensing performance, illustrating its potential for ultralow sensing of a wide range of analytes. In contrast, the NTA and BTA substrates demonstrated highly variable sensitivity, ranging from subfemtomolar detection to as high as nanomolar and even micromolar LOD (in BTA), suggesting a higher susceptibility to the analyte’s molecular structure and resulting surface chemistry kinetics.

The observed variations in detection limits across the various probe molecules can be reasonably interpreted based on broadly established adsorption mechanisms on nanostructured gold. Thiophenol derivatives such as ATP and CTP exhibit strong chemisorption to gold via Au–S bond formation, enabling efficient charge-transfer coupling and highly localized field interaction at nanogap regions, which typically results in superior enhancement and lower detection limits compared to physisorbing dye molecules such as R6G that rely primarily on weaker electrostatic or π–metal interactions.^32^ These charge transfer adsorption configurations in the thiophenol molecules also resulted in increased prominence of out-of-phase vibrational modes at higher ATP concentrations (Figure 5), indicating the increased contribution of charge-transfer and chemical enhancement as the surface approaches monolayer coverage (which has been reported to occur at nM concentrations for thiophenols).^32^ Meanwhile, differences in hotspot density, spatial distribution, and coupling strength among the three metasurface designs likely influence the adsorption configuration, molecular surface density, and electromagnetic enhancement experienced by R6G, thereby explaining the general comparatively higher detection thresholds.^18^ A more comprehensive discussion of the different substrates’ adsorption behavior, backed by quantitative derivations from experiments involving ATP (the analyte with the highest self-assembly propensity on gold substrates), is presented in Section S10.

It is important to note that the surface adsorption behaviors of the various substrates are also subject to the physicochemical properties of the analyte molecules governing their physisorption and chemical adsorption mechanisms. R6G, a typically physisorption-based SERS reporter reported a similar mathematical conformity (Langmuir-adherent on NTA and BTA; Freundlich’s on HCA) to the thiophenols, with fractional surface coverage ranging from 6% (HCA) to 30 (NTA) and 40 (BTA) at the respective calculated LOD values. These are expectedly higher than fractional surface coverage values at LODs obtained with the thiophenols. Additionally, HCA demonstrated a much less fractional surface coverage compared to NTA and BTA, with the physisorbing R6G dye, in contrast with the trend observed with self-assembling ATP, demonstrating the significance of analyte properties on surface adsorption and ultimately SERS activity.^41^ Therefore, while comprehensive ATP-substrate adsorption calculations were made to correct EF values obtained and characterize the electromagnetic enhancement of SERS activity by the respective substrates, these derivations cannot meaningfully be extrapolated to other analytes with different adsorption dynamics, due to their physicochemical properties not represented by ATP.^42–46^

The literature comparisons outlined in Table S6 offer a thorough framework for the design and manufacturing techniques, experimental performance, and application focuses of several SERS-based nanostructures in the literature. Several systems rely exclusively on simulation data, and the absence of experimental validation imposes practical constraints. Experimental EF values ranging from 10^7^ to 10^9^ and minimal detection limits have been attained on platforms such as microfluidic-based hybrid structures or silver-coated ordered nanogratings (UNGB arrays).^47^ Although these structures seem advantageous for biosensor applications, the intricacy of the fabrication process and the challenges associated with scaling up production are notable disadvantages. Likewise, TEPL cavity architectures including 2D materials (e.g., WSe_2_) can facilitate diverse photonic phenomena;^48^ however, performance criteria dictated by the Purcell effect, rather than the EF, constrain these structures to a narrow domain centered on quantum emission. Recent hybrid methodologies employing flexible PDMS-based structures and colloidal templates have also shown effectiveness, especially in chemical and biological studies, including flexible SERS sensors and pesticide detection.^49–51^ Nonetheless, these investigations have predominantly assessed performance by relative Raman signal enhancement, and numerical EF values have not been distinctly defined. In this context, the plasmonic metasurfaces created in our study demonstrated exceptional sensitivity, achieving a fM 4-ATP detection limit, while possessing an enhancement factor ∼10^7^, comparably with other structures documented in the literature.

Literature contains reports on the long-term stability of similar plasmonic structures. Zamboni et al. revealed that UV-ozone-coated copper nanoparticles maintained their plasmonic performance for a minimum of five months.^52^ Ding et al. emphasized the enduring chemical and structural durability of dynamic metasurfaces utilizing phase-change materials.^53^ Dielectric coating techniques have been documented to enhance the endurance of metasurfaces against oxidative degradation and mechanical damage.^54^ The results endorse the enduring efficacy of the structures employed in this investigation under suitable conditions.

## 4 Conclusion

In this study, three different plasmonic metasurface designs; BTA, HCA, and NTA integrated within a MIM architecture for SERS applications were designed, simulated, fabricated, and characterized. FDTD simulations showed that these metasurface structures exhibited strong LSPR and reached electric field strengths of *E* ^2^ ≈ 1600 at resonance wavelengths. This strong field concentration directly contributed to the enhancement of Raman signals. The combined evaluation of the three structures demonstrates that the proposed platforms are strong candidates for analyte determination at ultralow concentrations, with low detection limits reaching pico- and femtomolar levels and SERS amplification factors of the order of ∼10^6^ to 10^7^. This performance suggests their suitability not only for model molecules (R6G, 4-ATP, 4-CTP) but also for a wide range of applications, including pesticide detection, biomarker determination, environmental pollution analysis, and forensic analysis. Furthermore, the high structural regularity and controlled nanogap sizes provided by EBL and lift-off methods offer a more reliable, reproducible, and practical approach compared to colloidal or self-assembly based substrates. The preference of the EBL and lift-off methods in our study provides the fabrication of nanostructures in a controlled and repeatable manner. MIM-based systems can be operated in different wavelength ranges using different metal–insulator–metal combinations. When evaluated in terms of the production process, focusing on an optimized, more suitable MIM system for the application, rather than more complex and difficult-to-produce hybrid structures, is a significant advantage of this study.

Future studies aim to optimize spectral alignment with hybrid dielectric-metal stacks, develop scalable and cost-effective fabrication methods for large-area fabrication, and integrate these metasurfaces with portable and miniaturized spectroscopic platforms. Accordingly, it can be concluded that the MIM-based plasmonic metasurface designs presented here provide a strong foundation for the development of field-applicable, high-sensitivity, and multipurpose SERS sensor systems.

## Supplementary Material



## Notes

Comprehensive experimental section; detailed description of enhancement factor calculations; detailed information and figures outlining the reflectance-based design parametrization and optical characterization studies; representative unprocessed and preprocessed spectra; annotated spectra of the blank substrate surface, with assignments of the background peaks; and RSD calculations demonstrating signal reproducibility on the bare substrate (PDF)

## Abbreviations

ATP: aminothiophenol
BTA: Bowtie arrays
CCD: charge-coupled device
CTP: chlorothiophenol
DNA: DNA
EBL: electron beam lithography
EF: enhancement factor
FDTD: finite-difference time-domain
HCA: honeycomb array
LED: light-emitting diode
LOD: limit of detection
LSPR: localized surface plasmon resonance
MIM: metal–insulator–metal
NC: nanocube
NTA: nanotriangle array
PCNA: porcelain carbon nanowire array
R6G: rhodamine-6-G
SEM: scanning electron microscopy
SERS: surface-enhanced Raman scattering
SPR: surface plasmon resonance
TEM: transmission electron microscopy
UV: ultraviolet radiation.
