# Supplementary material for: Plasmon-Enhanced Raman Sensing with Metal–Insulator–Metal Metasurfaces
Source: ACS Appl Mater Interfaces. 2026 Apr 9;18(15):22246–60. doi: 10.1021/acsami.5c25818 (PMC13107380; doi:10.1021/acsami.5c25818)
Supplement: Supplementary file 1 [file am5c25818_si_001.pdf]

Supporting Information

Plasmon Enhanced Raman Sensing with Metal-Insulator-Metal Metasurfaces

Sümeyra Vural Kaymaz[1+], Fahd Khalid-Salako[2+], Hasan Sarıgül[3,] Beyza Nur Günaydın[2], Hasan Kurt[4,5,6,*], Meral Yüce[3*]

[1] Department of Molecular Biology, Genetics, and Bioengineering, Faculty of Engineering and Natural Sciences, Sabanci University, 34956, Istanbul, Türkiye

[2] Department of Materials Science and Nanoengineering, Faculty of Engineering and Natural Sciences, Sabanci University, 34956, Istanbul, Türkiye

[3] SUNUM Nanotechnology Research and Application Centre, Sabanci University, Istanbul, 34956, Türkiye

[4] Department of Bioengineering, Imperial College London, South Kensington Campus, London, SW7 2AZ, UK

[5] Department of Biomedical Engineering, School of Engineering and Natural Sciences, Istanbul Medipol University, Istanbul, 34810, Turkey

[6] Research Institute for Health Sciences and Technologies (SABITA), Istanbul Medipol University, Istanbul, 34810, Turkey

***Corresponding authors**: hasankurt@medipol.edu.tr and meralyuce@sabanciuniv.edu

+Equal Contribution.

Supporting Information

Table of Contents

S1. Experimental Section 3

S1.1. Simulation Studies 3

S1.2. Fabrication of Plasmonic Metasurfaces 3

S1.3. Reflection Measurements 4

S1.4. SERS Measurements 5

S1.5. Data Processing 5

S2. MIM Metasurfaces' Enhancement Factors 7

S3. 4-ATP Surface Coverage 11

S4. Reflectance Based–Design Parameterization Studies 13

S5. Refractive Index Study 15

S6. Representative Spectra Before and After Data Pre-Processing 18

S7. Representative Blank Substrate Spectrum 19

S8. RSD Comparison of Each Design 20

S9. Computational Optimization of the Dielectric Spacer Thickness 21

S10. ....Substrates Adsorption Behaviour 23

S11. ....Peak Assignment of Probe Molecules 25

S12.SERS Detection Performance of the Substrates Using Raman Reporters and Their Practicality 26

S13. ....Comparative SERS Performance of the Fabricated Metasurface Designs 31

S14. ....Comparative SERS Performance of Different Substrates with MIM 34

S15. ....Literature Comparison 35

References 39

Supporting Information

S1. Experimental Section

S1.1. Simulation Studies

The optical properties of the plasmonic arrays were simulated using Lumerical's FDTD software. The excitation light was selected as 500−1700 nm with a polarization in the x-axis. A perfectly matched layer was used in the z-axis direction, while an anti-symmetric boundary condition was used in the x- and symmetric y-axis directions. To determine the optical properties and electric field enhancement in the simulation, reflectance (R) and electric field monitors (XY and XZ) were placed at the appropriate locations for each array. A total field-scattered field (TFSF) source is used in the simulations of a single nano-antenna. The plane wave is initiated normally from the top of the unit cells. The TFSF source is placed outside the geometry to measure the reflectance cross-sections. Field enhancement (E/E_0) was determined as the ratio between the magnitudes of the electric fields on the placed monitor at the measured plane and the incident electric field. The optical properties of Au were modelled using our measurements, and Ti was selected from the Lumerical material database, along with Palm and Al from McPeak. SiN and SiO_2 films were modelled using a dispersion relation provided by Palik.

S1.2. Fabrication of Plasmonic Metasurfaces

Based on FDTD simulations, the aim is to develop periodic plasmonic SERS arrays with MIM architecture. Thermal evaporation was employed to coat metal layers, while the plasma-assisted chemical vapor deposition method was utilized to grow insulating layers. Before starting this procedure, it is important to clean and carefully dry the silicon wafer. For this purpose, silicon wafers were cleaned with piranha solution, and the surfaces were prepared for coating by applying oxygen plasma. For patterning, a dosing path was prepared using Beamer software, where parameters such as probe size, dose, and dosing path were defined. Using the thermal evaporation coating process, an Al layer of 100 nm thickness was first deposited on the silicon wafer. To enhance Al adhesion on the silicon wafer, 10 nm of Ti was coated via thermal evaporation at a rate of 0.5-0.8 Å/s, operating under a pressure of 3×10^{-6} Torr. Subsequently, a 100 nm layer of Al was deposited at a rate of 1.5-2 Å/s on top of the Ti layer, and then the system was vented. Throughout the coating process, substrates were rotated to achieve a uniform thickness distribution. Following the deposition of the first metal layer in the MIM structure, a 75 nm layer of SiO_2 was applied using a PECVD system for the BTA and NTA; and 150 nm Si_3N_4 for the HCA separately. Next, a photoresist layer was spin-coated with an e-beam resist, having a thickness of ~340 nm for bowtie and nanotriangle, and 360 nm for HCAs. After dosing with 280-290 µC/cm² using the EBL system, the resist layer

was developed. A 100 nm layer of Au was then thermally evaporated onto the patterned resist for the bowtie and nanotriangle, and 120 nm was deposited for the HCA. Finally, a lift-off process was used to fabricate the structure. The detailed surface morphology of the nanoarrays was visualized using a scanning electron microscope (SEM, Zeiss, Leo Supra VP 35).

S1.3. Reflection Measurements

A custom-made microspectroscopy setup collected reflection spectra of the surfaces. A parabolic mirror-based reflective collimator (Thorlabs, RC08SMA-P01) was used to collimate a fiber-coupled Tungsten-Halogen broadband light source in the microspectroscopy setup. A Keplerian beam expander with an iris at the focal point, and two achromatic doublets were then used to expand the light source. To ensure plane-wave illumination, the light beam was focused using an achromatic doublet (Thorlabs, AC254-150-AB, f = 150 mm) on the back-focal plane of long-working distance apochromatic objective lenses (Mitutoyo Plan Apo NIR 5X, 37.5 mm WD, 0.14 NA & Mitutoyo Plan Apo NIR 10X, 30.5 mm WD, 0.26 NA). The microspectroscopy setup's illumination and imaging/spectroscopy arms were divided using a plate beamsplitter (Thorlabs, BSW29R, 50:50). A joint tube lens (Thorlabs, TTL200-S8, f = 200 mm) was used in the imaging/spectroscopy arm to create the image on the spectrometer's slit plane as well as the imaging camera. Thorlabs CCM1-BS014, 50:50, a non-polarizing cube beamsplitter, was utilized to divide the spectroscopic and imaging arms. A monochrome CMOS camera (Basler, acA4112-30um, 12.3 MP, Sony IMX253 sensor) was used to capture the image in the imaging arm. An imaging spectrometer (PI, Isoplane SCT320) fitted with a spectroscopy CCD camera (Andor iVac 316 LDC-DD) was used in the spectroscopy arm to record the spectral response. A 2D motorized translation stage (Thorlabs, PLS-XY) in the xy-plane and a 1D motorized translation stage (Thorlabs ZFM2030) in the z-direction were used to achieve the sample's spatial translation.

Glycerol-based solutions were prepared to determine the refractive index sensitivity of substrates. To achieve this objective, glycerol-water solutions with concentrations varying from 1% to 50% were created as stock solutions, and the refractive index of each solution was measured and documented using a calibrated refractometer. Measurements were conducted utilizing a reflection configuration of a custom-made microspectroscopy setup. In each experiment, a 10 µL of the glycerol solution was applied to the metasurface to guarantee uniform distribution. A cover glass was subsequently affixed to the surface for measurements. This method systematically analyzed the optical refractive index responses of metasurfaces to glycerol solutions with varying concentrations.

S1.4. SERS Measurements

Before SERS measurements, the plasmonic metasurfaces underwent a rigorous cleaning procedure. The surfaces were submerged in ethanol, methanol, and isopropanol for 10 minutes each, subsequently undergoing sonication for 5–10 seconds in each solution. A 100 W plasma cleaning procedure was conducted for 30 seconds. The surfaces were dried using a clean air stream and subsequently stored in a desiccator to avert moisture and particle contamination till measurement. 1 M stock solutions were formulated for the analyte compounds. To achieve this, 4-ATP (CAS: 1193-02-8) and 4-CTP (CAS: 106-45-6) were solubilized in ethanol, whereas R6G (CAS: 989-38-8) was solubilized in ultrapure water to prepare stock solutions. Probe solutions of differing concentrations were prepared through suitable dilutions of the stock solutions. During the measurements, 15 µL of the relevant probe solution was applied to each metasurface, and the surface was thereafter covered with a cover glass to maintain continuity of measurement in the liquid phase.

The Raman excitation and signal acquisition were conducted utilizing a 60X microscope objective (CFI Plan Fluor 60x 0.85 NA, Nikon, Japan). The Raman system, details of which were previously published in our lab's study, [1] was configured for λ = 785 nm with a grating of 1200 lines/mm. The Raman signal was subsequently linked to a multimode optical fiber and transmitted to the spectrometer. We employed an aberration-free spectrograph (Isoplane SCT320, Princeton Instruments, USA) in conjunction with a back-illuminated CCD spectroscopic camera (iVac 316 LDC-DD, Andor, Northern Ireland) for the spectral detection of the Raman signal. The spectrum calibration of the spectrograph was sustained utilizing a Ne/Ar lamp (IntelliCal, Princeton Instruments, USA). The sample was imaged using a fiber-coupled broadband LED (MBB1L3, Thorlabs, Germany) and a color CMOS camera (acA3088-16gc, Basler, Germany). The laser power was regulated and observed with Cobolt MonitorTM software (Hubner Photonics, Sweden). The unprocessed Raman signal data was acquired utilizing Solis spectroscopy software (Andor, Northern Ireland). The spectral resolution of the in-house Raman spectrometer was less than 1 cm^{-1}. In the in-house Raman spectrometer, a laser power of 5 μW (connected to the fiber) was utilized, with a signal collection exposure period of 1 second and 30 accumulations. The Raman spectra of the probe molecules (*4-ATP*, *R6G*, and *4-ClTP*) were obtained in the liquid state at varying concentrations. A minimum of 10 measurements were conducted for each reporter to guarantee data accuracy and quality.

S1.5. Data Processing

The first step in the data analysis process is pre-processing, which aims to increase the visibility of peaks by removing background noise from the spectra. Pre-processing was done with the Python-based RamanSPy library (https://Ramanspy.readthedocs.io/en/latest/),[32] and

all spectra were processed through a pipeline of automatable and reproducible methods. For this purpose, a dedicated Raman Spectra Analyzer (RamanPlot) user interface was developed (**Figure S5-S6**) to facilitate more intuitive and user-friendly preprocessing. This interface allows multiple spectra to be loaded simultaneously and automatically processed using specified pipeline parameters. After selecting data files in the interface, users can adjust parameters such as the crop range, baseline correction method, denoise method, and filtering window length and polynomial order. Furthermore, the peak picking feature can be activated in the interface, and threshold values (prominence, minimum width, minimum height, minimum distance, etc.) for peak detection can be flexibly defined by the user. The lower section of the interface offers various options for exporting processed or unprocessed data, detected peaks, and individual spectra. This not only speeds up the pre-processing process but also ensures a uniform and reproducible data processing flow across all concentration series.

The Whitaker–Hayes despiking algorithm was applied to eliminate narrow, high-intensity noise signals originating from cosmic rays in the spectra. Subsequently, baseline correction based on IARPLS (Improved Asymmetrically Reweighted Penalized Least Squares) was selected for the baseline correction to preserve the peaks and normalize the non-peaked areas. Smoothing and denoising were then performed using the Savitzky–Golay filtering method (window length: 8, polynomial degree: 2) to preserve the location and shape of the Raman peaks while reducing high-frequency noise. All reporter spectra underwent pre-processing using the Python pipeline. Replicate spectra were batch-processed, following the described pre-processing pipeline. Post-processing involved data normalization against a consistent background peak, empirically observed at ~1010, 1160, or 1265 cm^{-1}, to reduce variability arising from heterogenous sample distribution on the sensor surfaces, temporal artefacts like sample drying, and other factors. This enabled a reliable comparison of intensity differences between spectra obtained at different concentrations. Processed replicate spectra were averaged and presented, alongside calibration curves obtained from plots of characteristic peak heights against concentration on a log scale, fitted with a logistic function. Limits of Detections were calculated as concentrations on the calibration curves, corresponding to $[\bar{y} + (3.33 \times \partial)]$, where $\bar{y}$ is the maximum normalized intensity within a 10 cm^{-1} window around the characteristic peak, and ∂ is the standard deviation at $\bar{y}$; of the blank (zero) sample spectra respectively.[2,3]

S2. MIM Metasurfaces' Enhancement Factors

SERS signal depends on the laser power, excitation time, and number of molecules excited. Since direct comparison at different excitation wavelengths can lead to errors, SERS and reference Raman measurements for the same wavelength were performed with fixed parameters. In this way, the EF can be used to evaluate SERS performance in different conditions. A well-accepted and widely used definition is the ratio of the SERS intensity (I_{SERS}) of a molecule to the normal Raman intensity (I_{REF}) of the same molecule in the absence of the substrate with the following formula [4]:

$$\text{EF} = (\boldsymbol{I_{SERS}}/\boldsymbol{N_{SERS}})/(\boldsymbol{I_{REF}}/\boldsymbol{N_{NORM}}) \quad (S1)$$

Where $\boldsymbol{N_{SERS}}$ and $\boldsymbol{N_{NORM}}$ are the number of molecules detected in the SERS and normal Raman measurements of SERS and reference samples at 1077 cm^{-1} for 4-ATP molecule. Following this definition, it is assumed, initially, that the probe molecules adsorb as a full monolayer on the substrate surface (fractional surface coverage terms are applied at lower concentrations to correct for this assumption). For all measurements, an x60 objective with a numerical aperture of 0.85 was employed to conduct Raman measurements.

Following an approximation used in previous SERS publications,[5–7] the numerical-aperture-limited Gaussian beam model was applied. In this approach, the beam waist radius is estimated as:

$$w_0 \approx \frac{2\lambda}{\pi \text{NA}} \quad (S2)$$

with the Rayleigh range given by:

$$z_R = \frac{\pi w_0^2}{\lambda} \quad (S3)$$

The number of molecules probed in the reference Raman measurement (N_{REF}) was estimated from the effective excitation volume, which was derived using Gaussian beam optics. The laser focus was modeled as a diffraction-limited Gaussian beam, with the beam waist diameter calculated as: $w = \frac{4\lambda}{\pi \text{NA}} = 1.176\ \mu\text{m}$

The corresponding depth of focus was obtained as:

$$z = \frac{2\pi w^2}{\lambda} = 11.07 \quad (S4)$$

The effective focal volume was then calculated using:

$$V_{\text{eff}} = \left(\frac{\pi}{2}\right)^{3/2} w^2 z = 30.15\text{um}^3 \quad (S5)$$

Supporting Information

Using a 1 M aqueous solution of 4-ATP and Avogadro's constant, the number of molecules contributing to the reference Raman signal was calculated as:

$$N_{\mathrm{REF}} = V_{\mathrm{eff}} \times C \times N_A = 1.81 \times 10^{10} \qquad (S6)$$

For the SERS measurement, the number of molecules (N_{SERS}) was estimated, assuming monolayer adsorption of 4-ATP on the metasurface. The laser spot area was calculated as;

$$A_{\mathrm{laser}} = \pi\left(\frac{w}{2}\right)^2 = 1.09\ \mathrm{um}^2 \qquad (S7)$$

Considering a molecular footprint of 4-ATP of

$$2.20 \times 10^{-5}\ \mathrm{um}^2$$

per molecule, the number of molecules within the laser spot was estimated as:

$$N_{\mathrm{SERS}} = \frac{A_{\mathrm{laser}}}{A_{\text{4-ATP}}} = 4.94 \times 10^4 \qquad (S8)$$

Based on this approach EF values calculated as follows;

**BTA (10^{-11})**	**BTA (10^{-9})**	**BTA (10^{-7})**
2.81×10^6	6.01×10^6	1.62×10^7
**NTA (10^{-11})**	**NTA (10^{-9})**	**NTA (10^{-7})**
5.87×10^6	8.80×10^6	1.59×10^7
**HCA (10^{-11})**	**HCA (10^{-9})**	**HCA (10^{-7})**
4.56×10^6	4.98×10^6	6.55×10^6

For verification purposes, the focal parameters were also estimated using the input beam diameter at the back aperture of the infinity-corrected microscope objective. The beam waist is approximated as:

$$w_0 \approx \frac{4\lambda f_{\mathrm{obj}}}{\pi D_{\mathrm{in}}} \qquad (S10)$$

Where $f_{\mathrm{obj}} = L/M$is the effective focal length of the objective. The Rayleigh range and focal volume were subsequently calculated using the same standard Gaussian beam expressions. For comparison, the previous calculation yielded a larger excitation volume and a correspondingly higher number of reference molecules ($N_{\mathrm{REF}} = 1.81 \times 10^{10}$). All other parameters were identical between the two calculations. This approach directly links the focal volume to the experimentally measured beam size and in the general discussion of the study is done based on this approach. It was derived using an input-beam–limited Gaussian beam model, where the beam waist was calculated from the input beam diameter at the objective back aperture ($D = 5$ mm) and the effective focal length of the objective ($f_{\mathrm{obj}} = 3.33$ mm). This yielded a beam waist of $w_0 = 6.67 \times 10^{-7}$m and a Rayleigh range of $z_R = 1.78 \times 10^{-6}$m. Using these values, the effective focal volume was calculated as: $V_{\mathrm{eff}} = 1.55$ um^3

Based on this revised focal volume, the number of molecules contributing to the reference Raman signal was recalculated as:

$$N_{\mathrm{REF}} = 9.36 \times 10^{8}$$

Based on this approach EF values calculated as follows;

**BTA (10^{-11})**	**BTA (10^{-9})**	**BTA (10^{-7})**
4.53x10^5	9.67x10^5	2.60x10^6
**NTA (10^{-11})**	**NTA (10^{-9})**	**NTA (10^{-7})**
9.44x10^5	1.42x10^6	2.56x10^6
**HCA (10^{-11})**	**HCA (10^{-9})**	**HCA (10^{-7})**
7.38x10^5	8x10^6	5.21x10^6

Adopting a third approach, the focal spot size is experimentally determined as the 1/e² intensity diameter D_{1/e^2}, the beam waist is defined as $w_0 = \frac{D_{1/e^2}}{2}$. The Rayleigh range is calculated using the standard Gaussian beam relation:

$$z_R = \frac{\pi w_0^2}{\lambda} \qquad (S11)$$

and the effective focal volume is given by:

$$V_{\mathrm{focal}} = \left(\frac{\pi}{2}\right)^{3/2} w_0^2 z_R \qquad (S12)$$

Based on this revised focal volume, the number of molecules contributing to the reference Raman signal was recalculated as:

$$N_{\mathrm{REF}} = 6.29 \times 10^{10}$$

Based on this approach EF values calculated as follows;

**BTA (10^{-11})**	**BTA (10^{-9})**	**BTA (10^{-7})**
2.10x10^5	4x10^5	1.21x10^6
**NTA (10^{-11})**	**NTA (10^{-9})**	**NTA (10^{-7})**
4.3x10^5	6.55x10^5	1.19x10^6
**HCA (10^{-11})**	**HCA (10^{-9})**	**HCA (10^{-7})**
3.42x10^5	3.71x10^5	2.41x10^6

A comparison of the simulated and experimental EF values (based on the objective back aperture input beam diameter approach) is presented in **Table S1**.

Table S1. Comparison of theoretical and experimental EF values.

**Theoretical EF**	**Experimental EF**

	$\lvert E_{loc\,(excitation)}\rvert/\lvert E_0\rvert^2 \times \lvert E_{loc\,(excitation)}\rvert/\lvert E_0\rvert^2$	$(I_{SERS}/N_{SERS})/(I_{REF}/N_{NORM})$		
Design	*Simulation*	*C_{SERS} 10^{-11} M*	*C_{SERS} 10^{-9} M*	*C_{SERS} 10^{-7} M*
BTA-MIM	2.25×10^6	4.53×10^5	9.67×10^5	2.60
HCA-MIM	1.3×10^7	7.38×10^5	8.0×10^5	2.56
NTA-MIM	4.95×10^6	1.01×10^6	1.42×10^6	5.21

S3. 4-ATP Surface Coverage

Measurements were conducted with 15 µL aliquot spot volumes; assuming a 0.22 nm^2 surface area coverage by a single molecule / molecular footprint:[8]

For SERS measurements

Total moles in 10^{-11} M = 1.5 × 10^{-16} mol

Number of molecules = 9.033 × 10^7 (NA = 6.022x10^{23})

Total surface area coverage by 4-ATP molecules = 1.81 × 10^{-5} mm^2

For normal Raman measurement

Total moles in 1 M = 1.5 × 10^{-5} mol

Number of molecules = 9.033 × 10^{18}

Total surface area coverage by 4-ATP molecules = 1.81 × 10^6 mm^2 (1.81 × 10^4 cm^2)

Limits of the monolayer coverage assumption

Each 1 cm^2 SERS chip contains 4 active areas, while each active area contains 24 NTA and BTA arrays; and 16 in the case of HCA arrays.

- **HCA**

  Each 60 µm × 60 µm HCA array contains roughly 19044 Au-hexagons, with a total surface area:

  ≈ (19044 × 0.15 μm^2)

  Therefore, the total gold surface area on a single HCA chip:

  SA_{HCA} = 4 × 16 × (19044 × 0.15 μm^2) = 0.18 mm^2

- **NTA:**

  Each 60 µm × 60 µm NTA array contains roughly 2900 Au-nanotriangles, with a total surface area:

  ≈ (2900 × 0.18 μm^2)

  The total gold surface area on a single NTA chip:

  SA_{NTA} = 4 × 24 × (2900 × 0.18 μm^2) = 0.05 mm^2

- **BTA:**

  Each 60 µm × 60 µm BTA array contains roughly 5250 Au-nanotriangles, with a total surface area:

  ≈ (5250 × 0.172 μm^2)

  Total gold surface area on a single BTA chip:

  SA_{BTA} = 4 × 24 × (5250 × 0.172 μm^2) = 0.09 mm^2

Accounting for the relative affinity of 4-ATP molecules for the gold-covered surfaces, the calculated limits of monolayer coverage are obtained thus:

Number of Molecules = 8.18 × 10^{11} (HCA); 2.27 × 10^{11} (NTA); and 4.1 × 10^{11} (BTA) respectively,

Corresponding to 1.32×10^{-12}; 3.77×10^{-13}; and 6.81×10^{-13} mols and 19.73 μM; 5.7 μM; and 10.22 μM; in a 15 μL sample aliquot on HCA, NTA, and BTA respectively.

Theoretically, up to ~5 μM, 10 μM, and 20 μM 4-ATP concentrations would be required to exceed monolayer Au-surface adsorption on NTA, BTA, and HCA respectively, during SERS measurements with the reported experimental proedure. By implication, a fractional surface coverage correction would be required when working at concentrations below these estimations.

S4. Reflectance Based–Design Parameterization Studies

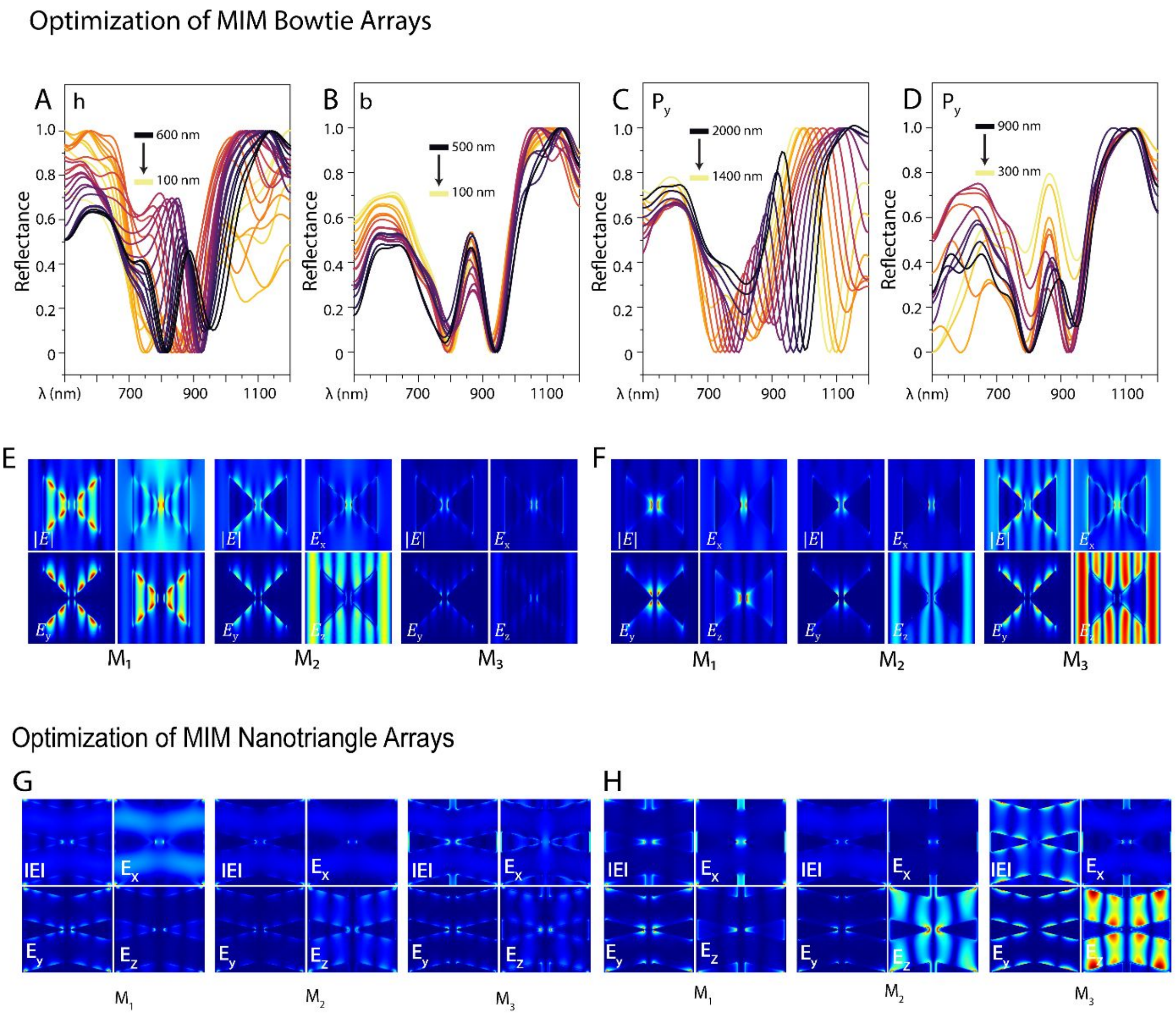

Figure S1. Optimization of MIM-based bowtie and nanotriangle nanoarrays. (A–D). Simulated reflectance spectra illustrating the effect of key geometric parameters on the optical response of bowtie nanoarrays: (A) triangle height (h), (B). base length (b), (C). horizontal periodicity (P_x), and (D). vertical periodicity (P_y). Variations in these parameters modulate resonance position and intensity, enabling spectral tuning of the dual LSPR modes. (E–F). Electric field distribution maps at resonance wavelengths for the optimized bowtie structures, showing |E| and vector components (E_x, E_y, E_z) at different monitored planes (M_1–M_3). The results highlight strong dipolar coupling and pronounced field localization within the nanogap and at metal–dielectric interfaces. (G–H). Corresponding electric field distributions for nanotriangle arrays, demonstrating field confinement behavior and comparatively different hotspot distributions across monitored planes.

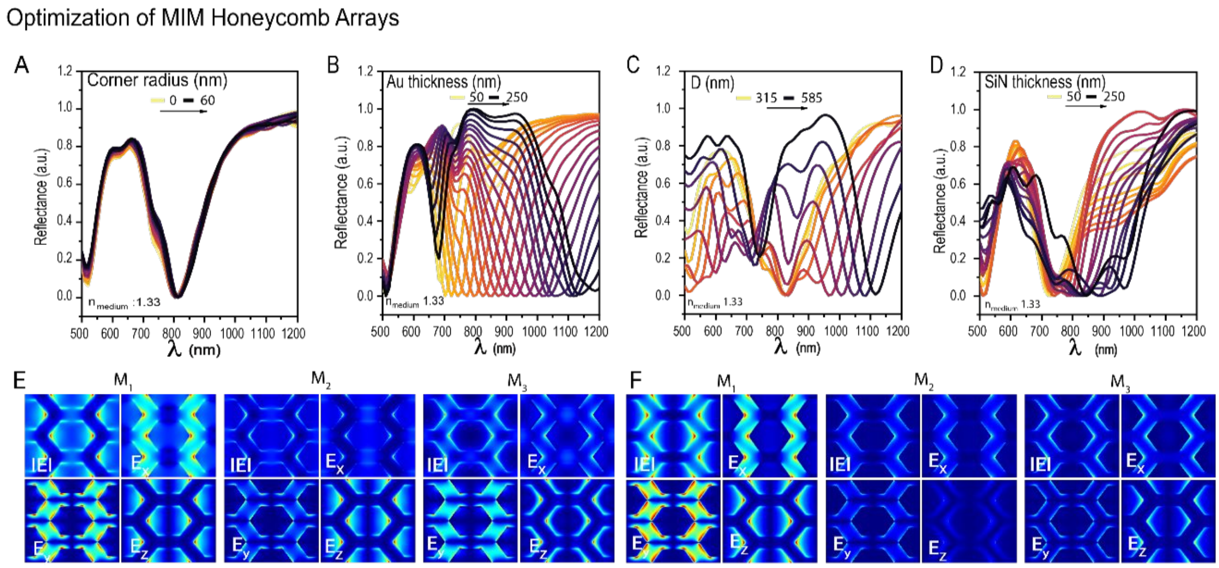

Figure S2. Optimization of MIM-based honeycomb nanoarrays. (A–D). Simulated reflectance spectra illustrating the effect of key structural parameters on the optical response of the honeycomb nanoarrays: (A). corner radius, (B). Au thickness, (C). hexagon diameter, and (D). Si_3N_4 thickness. Variations in these parameters modulate both the resonance wavelength and intensity, enabling controlled tuning of the dual LSPR modes through geometry and layer dependent optical coupling within the MIM architecture. (E–F) Electric field distribution maps at the resonance wavelengths for the optimized honeycomb structures, showing vector components (E_x, E_y, E_z) across different monitored planes. The results reveal strong field localization along the hexagonal edges and junction regions, with enhancement at the metal–dielectric interface. Distinct modal profiles indicate efficient plasmon confinement and geometry-dependent hotspot formation within the honeycomb lattice.

S5. Refractive Index Study

Table S2. Refractive index calculations for glycerol-water solutions at varying glycerol concentrations (wt.%) based on the Brix values at 20°C. Data demonstrate the glycerol concentration, Brix value, and refractive index, providing essential information for the optical characterization of glycerol-water concentrations.

Glycerol Concentrations (wt.%)	Brix Value (20°C)	n_{medium} $n=1.332+(0.0015\times Brix)+(2.8\times10^{-6}\times Brix^2)$
1	1	1.333503
3	2	1.335011
5	5	1.33957
10	10	1.34728
20	18.5	1.360708
30	25	1.37125
40	33	1.384549
50	39.5	1.395619

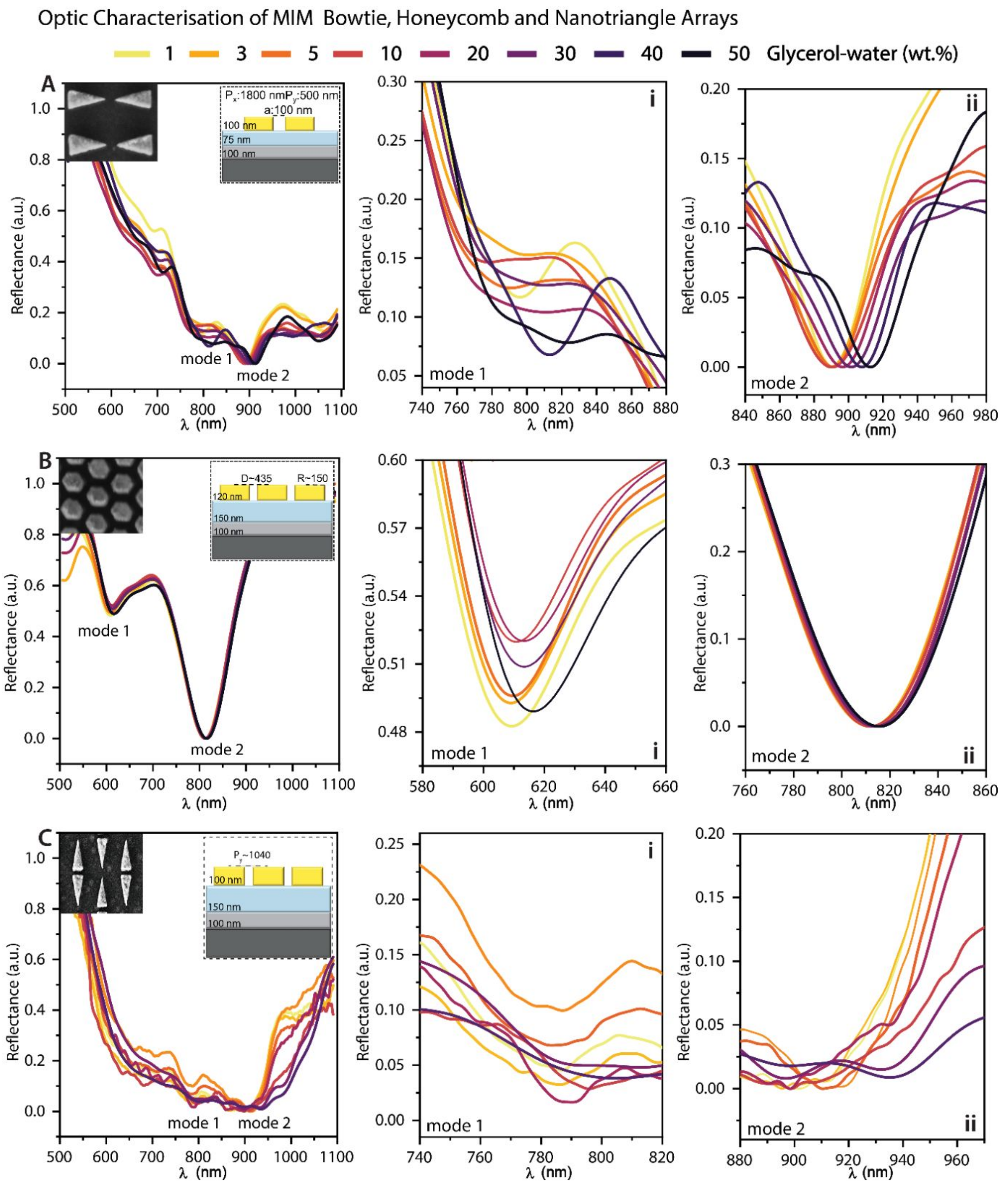

Figure S3. Optical characterization of MIM BTA (A). HCA (B). and NTA (C). in glycerol–water solutions. Glycerol concentrations ranging from 1 to 50 wt.% were prepared, and the corresponding refractive indices were determined using a refractometer. Reflection spectra of the optimized metasurface arrays were recorded under these conditions, and two primary resonance modes (Mode 1 and Mode 2) are presented for each array design.

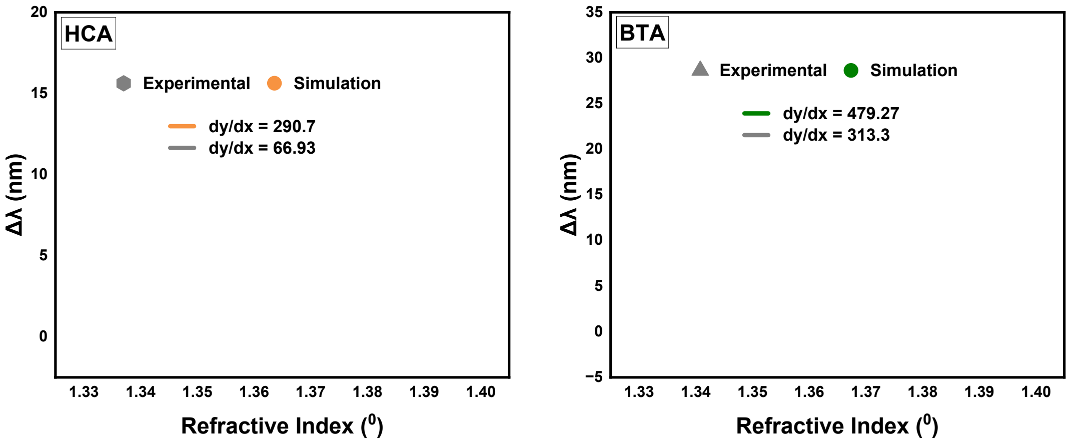

Figure S4. Comparisons of refractive index sensitivity of LSPR modes of interest on the HCA and BTA MIM metasurfaces.

S6. Representative Spectra Before and After Data Pre-Processing

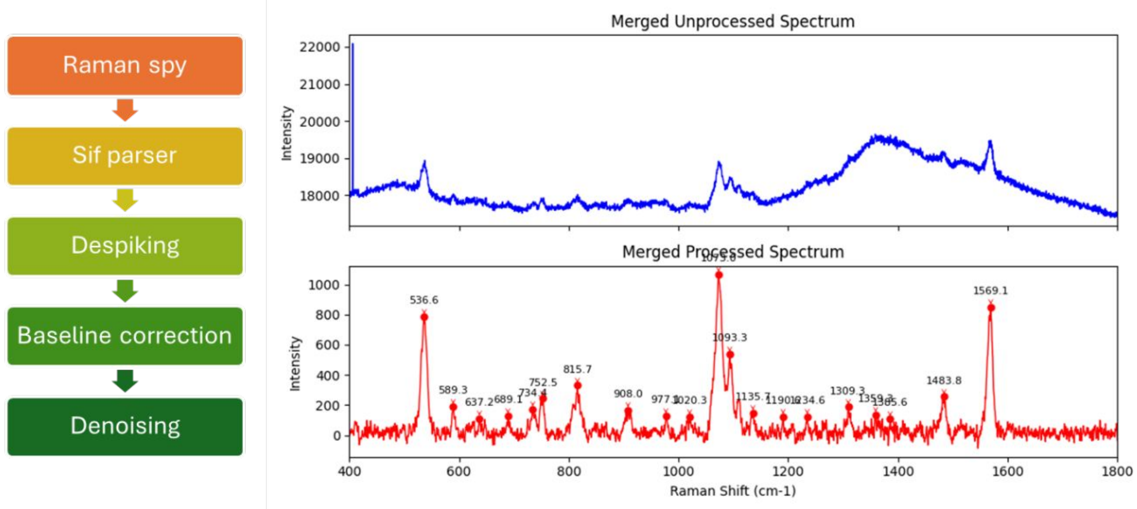

Figure S5. Data pre-processing steps and representative spectra before and after data pre-processing.

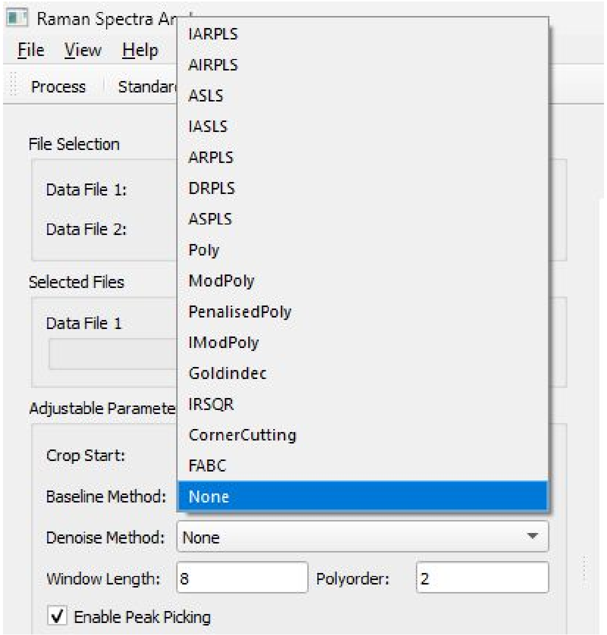

Figure S6. Available baseline correction methods in the Raman data pre-processing workflow.

S7. Representative Blank Substrate Spectrum

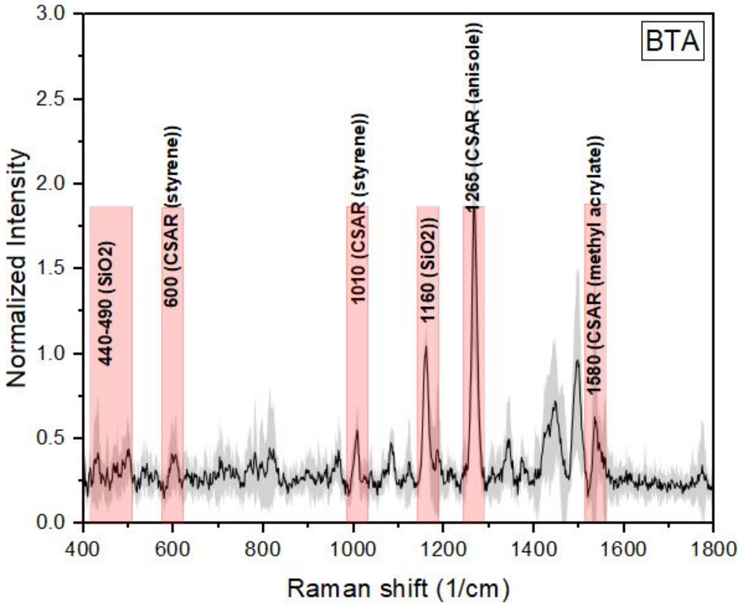

Figure S7. Representative Blank Substrate Spectrum[9–13]

S8. RSD Comparison of Each Design

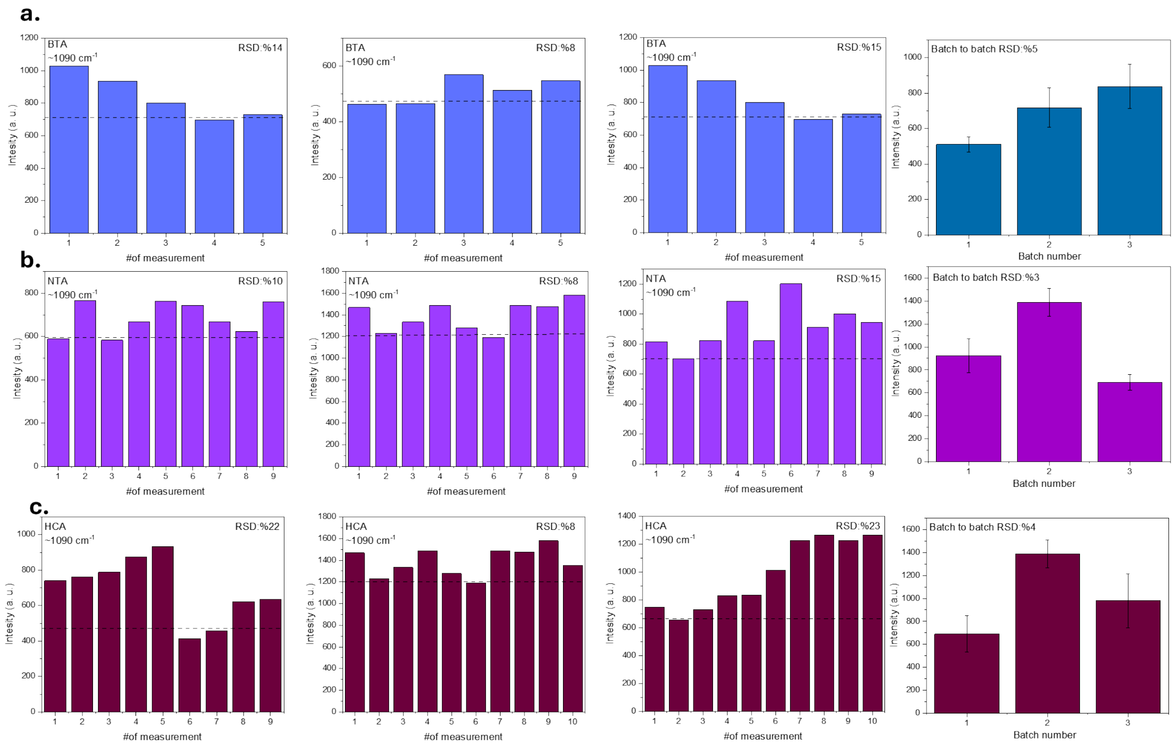

Figure S8. Precision and repeatability analysis of the SERS response obtained from the three metasurface architectures. Bar plots represent the mean SERS intensities extracted from characteristic Raman band of 4-ATP (~1090 cm-1), while error bars indicate the standard deviation calculated from repeated measurements at different spots and/or batches.

S9. Computational Optimization of the Dielectric Spacer Thickness

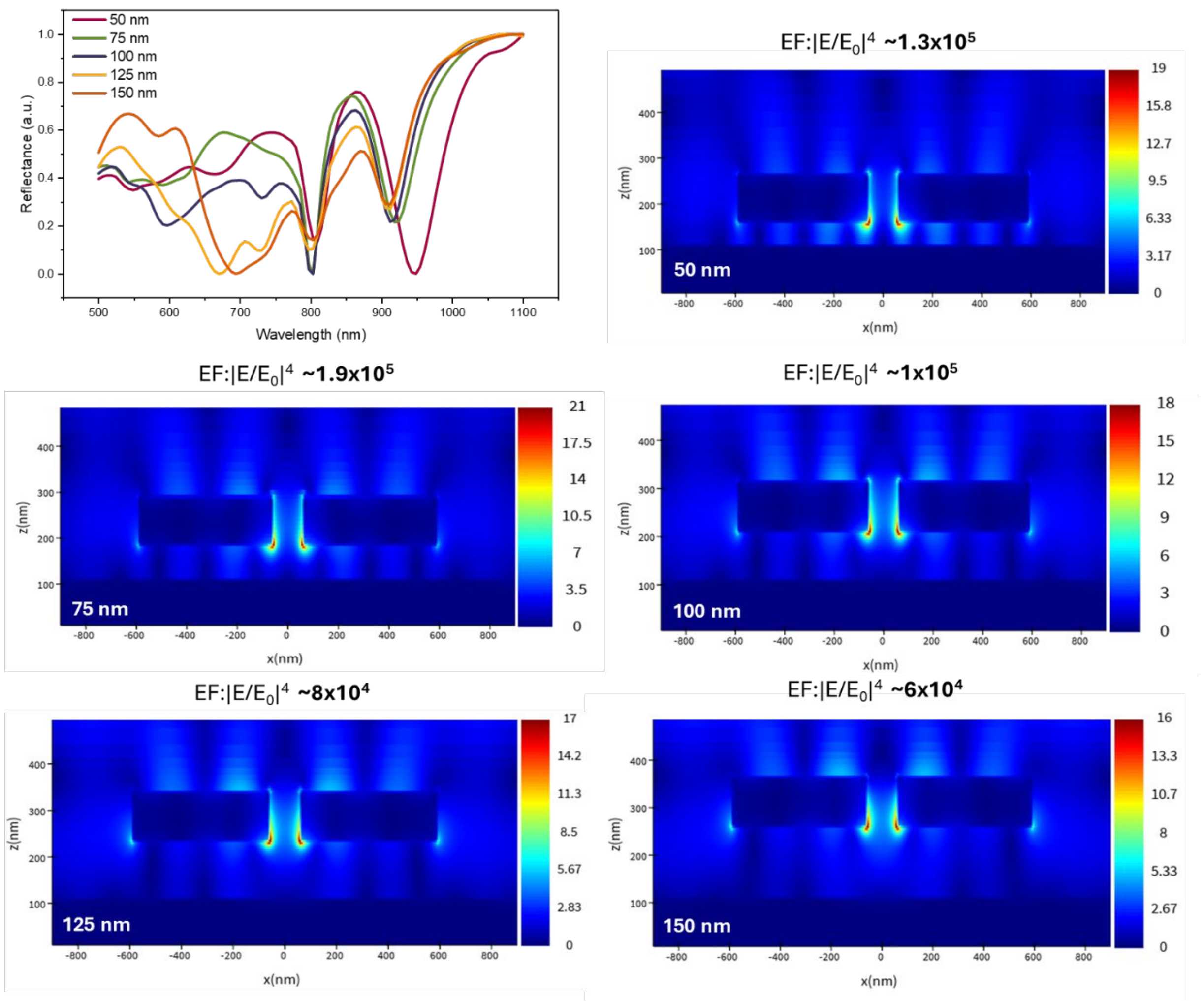

Figure S9. FDTD-simulated electric field distributions ($|E|/|E_0|$) of the MIM nanoplasmonic substrate at the resonant wavelength around 803 nm, showing the dependence of local field enhancement on dielectric spacer thickness in BTA. Variations in spacer thickness significantly modify the field confinement within the dielectric layer and plasmonic hotspots due to the coupling between localized plasmons on the top nanostructures, their mirror images, and back-reflector–supported polaritonic modes. The simulations were performed using a uniform 5 nm spatial mesh (coarser than the typically employed 1 nm mesh) while still adequately resolving the thickness-dependent plasmonic coupling behavior.

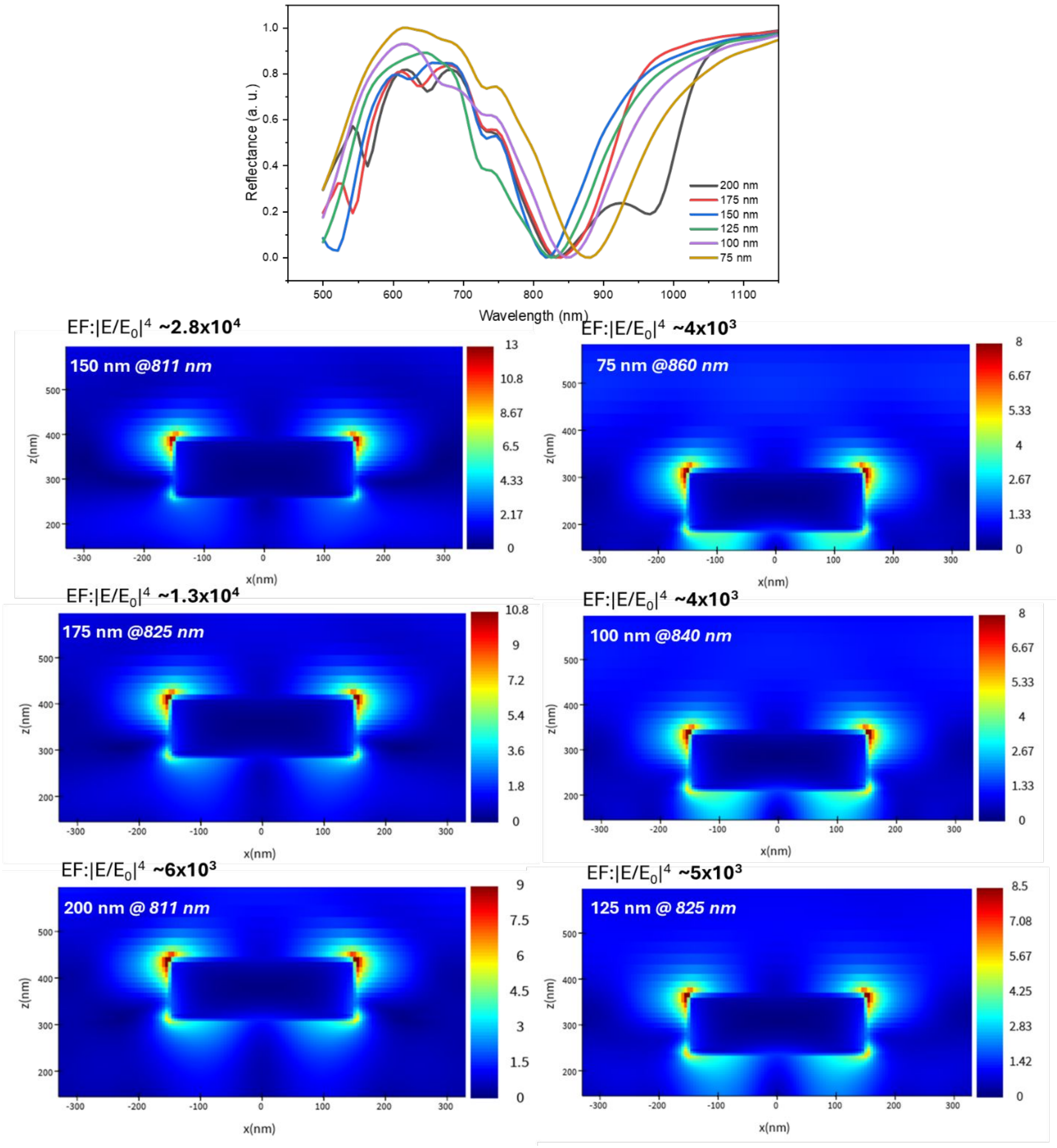

Figure S10. FDTD-simulated electric field distributions ($|E|/|E_0|$) of the MIM nanoplasmonic substrate at the resonant wavelength of each design where the E field is maximum, showing the dependence of local field enhancement on dielectric spacer thickness in HCA. Variations in spacer thickness significantly modify the field confinement within the dielectric layer and plasmonic hotspots due to the coupling between localized plasmons on the top nanostructures, their mirror images, and back-reflector–supported polaritonic modes. The simulations were performed using a uniform 5 nm spatial mesh (coarser than the typically employed 1 nm mesh) while still adequately resolving the thickness-dependent plasmonic coupling behavior.

S10. Substrates Adsorption Behaviour

The mechanism underlying SERS sensing is conceptually the generation of enhanced Raman scattering events, due to vibrational modes of molecules present —or more accurately, adsorbed— at specific nanoplasmonic hotspot sites on the substrates. Simply, the SERS signal obtained is a function of the nanoplasmonic enhancement at these hotspots, as well as the local surface site coverage, by the target molecules. Mathematically, this can be represented by:

$$I_{SERS} \propto \sum_i \theta_i |E_i|^4$$

Where $|E|^4$ represents the local field enhancement factor, while θ is the fractional local site coverage, over all plasmonic hotspots *i*. Consequently, quantitative derivations of the surface adsorption dynamics over all three metasurfaces can be obtained from the I_{SERS} – concentration plots, providing important insights into the adsorption regimes governing their SERS activities,[14,15] as well as means to correct analytical EF calculations at lower concentrations where monolayer adsorption is not assured.

Accordingly, normalized SERS intensities (I_{SERS}) against concentration plots obtained with ATP on the three substrates were fitted with adsorption isotherms, to obtain adsorption parameters including I_{SAT} which quantifies the extrapolated I_{SERS} at full surface saturation; adsorption constants, and similar parameters.

The equations of the Langmuir's and Freundlich's isotherms adopted are presented below:

Freundlich's Isotherm: $\theta = K_F[ATP]^{1/n}$ *(S13)*

Langmuir's Isotherm: $\theta = \frac{K_L[ATP]}{1+K_L[ATP]}$ *(S14)*

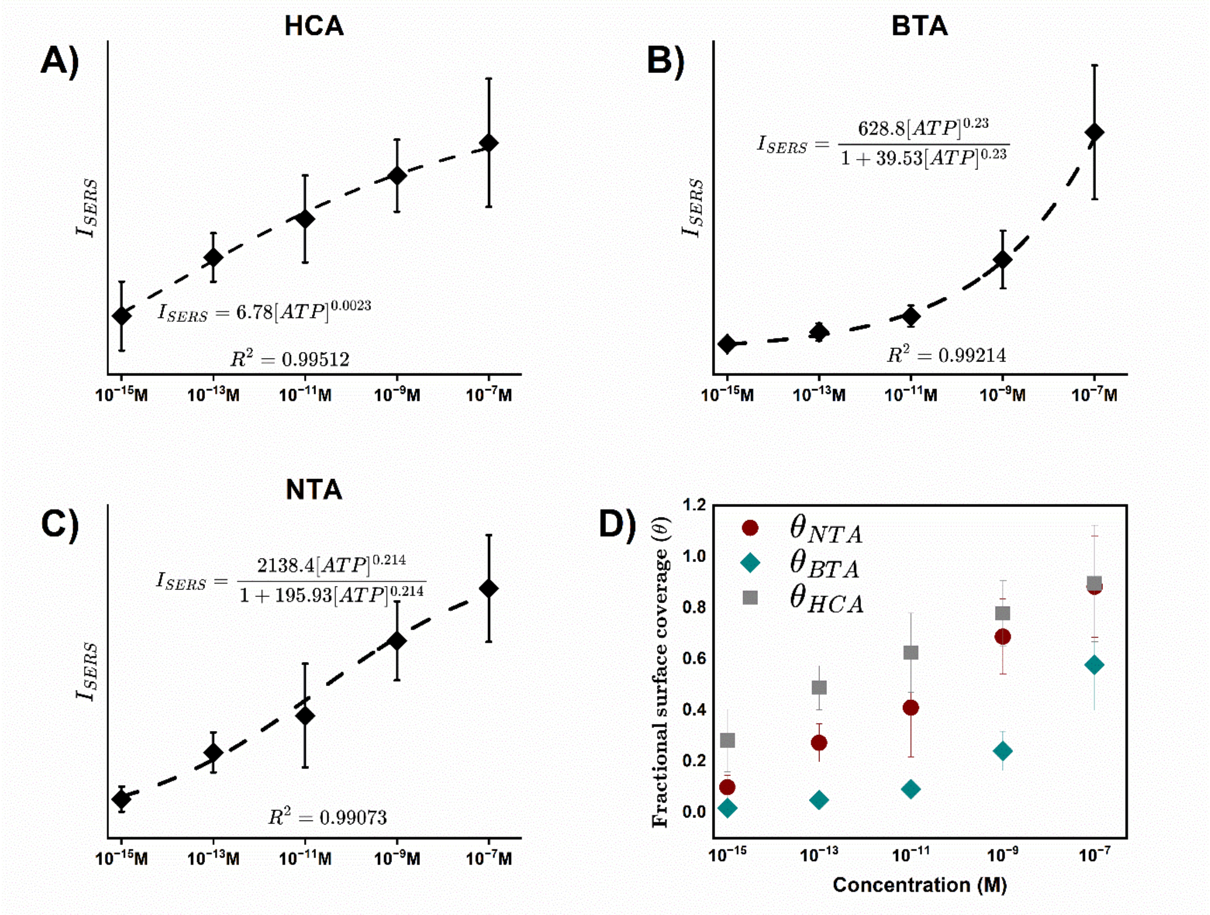

Figure S11. Plots of dependence of I_{SERS} on ATP concentration on A) HCA; B) BTA; and C) NTA MIM substrates, fitted with the Freundlich's (A) and Langmuir's (B and C) isotherms. D) Plots of fractional surface coverage (θ) against concentration for ATP on the NTA, BTA, and HCA substrates, obtained by normalizing I_{SERS} at each concentration against the I_{SAT} values obtained from the isotherm functions.

Notably, while the BTA and NTA curves were well fitted with a Langmuir isotherm function,[16] a good fit was achieved with the HCA curve only with a Freundlich's function.[17] This suggests a significant deviation of the adsorption behaviour on HCA, from the homogeneity assumption inherent in the Langmuir's isotherm.[18] The Freundlich's isotherm deviates from Langmuir's model by assuming surface heterogeneity quantified on a scale of 0 to 1. The Freundlich's isotherm also assumes multilayer adsorption and does not impose a saturation limit, as it only quantifies a change in adsorption rate as lower affinity adsorption sites dominate at higher analyte concentrations. The presence of heterogenous hotspots on the HCA is in order with its relative geometric complexity and consequent presence of several distinct plasmonic coupling regions like hexagonal corners, walls, edges, and small interstitial voids. Experimentally, this surface heterogeneity could also explain the especially noisy (high variance) background

signals obtained on HCA, compared to the other substrates (**Figure S14**). The fitting parameters obtained for ATP on the various substrates are presented in **Table S3**.

Table S3. Adsorption isotherm parameters obtained from the fitting functions presented in **Figure S11**.

Substrate	Isotherm type	Equation	Parameters	R^2
HCA-MIM	Freundlich	$\theta = K_F[ATP]^{1/n}$	$I_{\sim SAT}$ (I_{SERS} as surface approaches saturation) = 6.78<br>$1/n$ (empirical coefficient of surface heterogeneity: $0 \leq \frac{1}{n} \leq 1$) ≈ 0.00225	0.99512
NTA-MIM	Langmuir	$\theta = \frac{K_L[ATP]}{1 + K_L[ATP]}$	I_{SAT} (I_{SERS} at full surface saturation) = 10.91<br>K_L (Langmuir's adsorption constant) = 195.93 M^{-1}	0.99073
BTA-MIM			I_{SAT} = 15.91<br>K_L = 39.53 M^{-1}	0.99214

S11. Peak Assignment of Probe Molecules

Table S4. Raman peak positions and their vibrational assignments of probe molecules used in this study.

Raman peak position (cm^{-1})	Probe molecules	Vibrational assignments	Ref.
**610/600**	R6G	C-C-C in-plane bending	19
**770**	R6G	C-H out-of-plane bending	
**1363**	R6G	Aromatic C-C stretching	
**1309**	R6G	In-plane xanthene ring breathing; N-H bending; CH wagging	20
**1499**	R6G	Xanthene ring stretching; C-N stretching; C-H bending; N-H bending	

**1090**	4-ATP	C-S stretching + C-H bending	[8]
**1590**	4-ATP	Aromatic C=C stretching	
**536**	4-CTP	C-Cl stretching	[21]
**510**	4-CTP	Ring deformation	
**1074**	4-CTP	C-H in-plane bending	
**1095**	4-CTP	C-S stretching	
**1100**	4-CTP	C-H stretching	
**1568**	4-CTP	Aromatic C=C stretching	[8]

S12. SERS Detection Performance of the Substrates Using Raman Reporters and Their Practicality

The surface performance was further evaluated using both R6G and 4-CTP as Raman reporter molecules. A detailed discussion of these results is provided in the main manuscript.

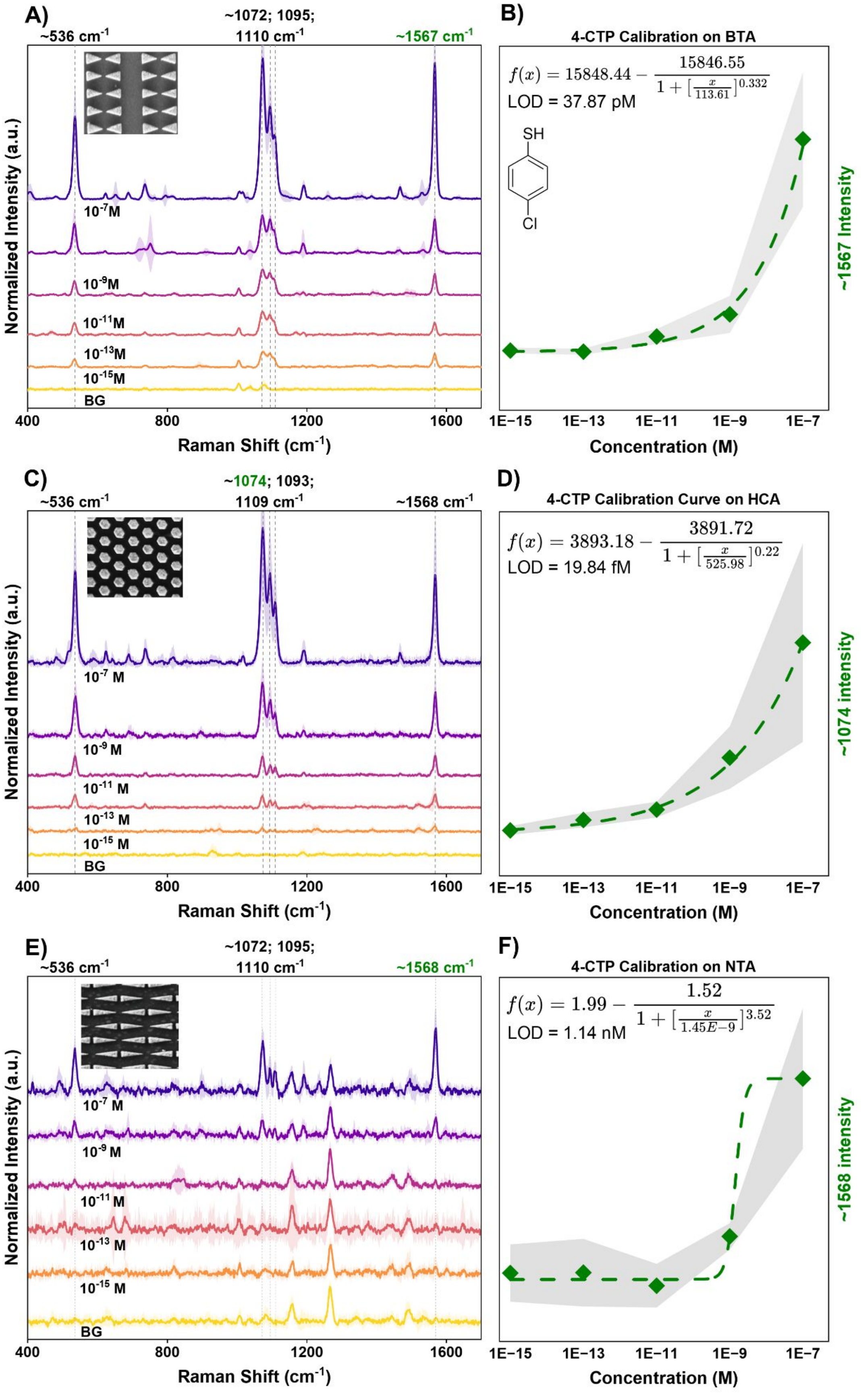
A)
~536 cm-1
~1072; 1095;
1110 cm-1
~1567 cm-1
Normalized Intensity (a.u.)
10-7M
10-9M
10-11M
10-13M
10-15M
BG
400
800
1200
1600
Raman Shift (cm-1)
B)
4-CTP Calibration on BTA
$f(x) = 15848.44 - \frac{15846.55}{1+[\frac{x}{113.61}]^{0.332}}$
LOD = 37.87 pM
SH
Cl
~1567 Intensity
1E−15
1E−13
1E−11
1E−9
1E−7
Concentration (M)
C)
~536 cm-1
~1074; 1093;
1109 cm-1
~1568 cm-1
Normalized Intensity (a.u.)
10-7 M
10-9 M
10-11 M
10-13 M
10-15 M
BG
400
800
1200
1600
Raman Shift (cm-1)
D)
4-CTP Calibration Curve on HCA
$f(x) = 3893.18 - \frac{3891.72}{1+[\frac{x}{525.98}]^{0.22}}$
LOD = 19.84 fM
~1074 intensity
1E−15
1E−13
1E−11
1E−9
1E−7
Concentration (M)
E)
~536 cm-1
~1072; 1095;
1110 cm-1
~1568 cm-1
Normalized Intensity (a.u.)
10-7 M
10-9 M
10-11 M
10-13 M
10-15 M
BG
400
800
1200
1600
Raman Shift (cm-1)
F)
4-CTP Calibration on NTA
$f(x) = 1.99 - \frac{1.52}{1+[\frac{x}{1.45E-9}]^{3.52}}$
LOD = 1.14 nM
~1568 intensity
1E−15
1E−13
1E−11
1E−9
1E−7
Concentration (M)

Figure S12. 4-CTP SERS Sensing on the MIM substrates. A, C & E. Representative spectra obtained on the BTA, HCA, and NTA respectively. B, D & F. 4-CTP Calibration curves on the BTA, HCA, and NTA respectively.

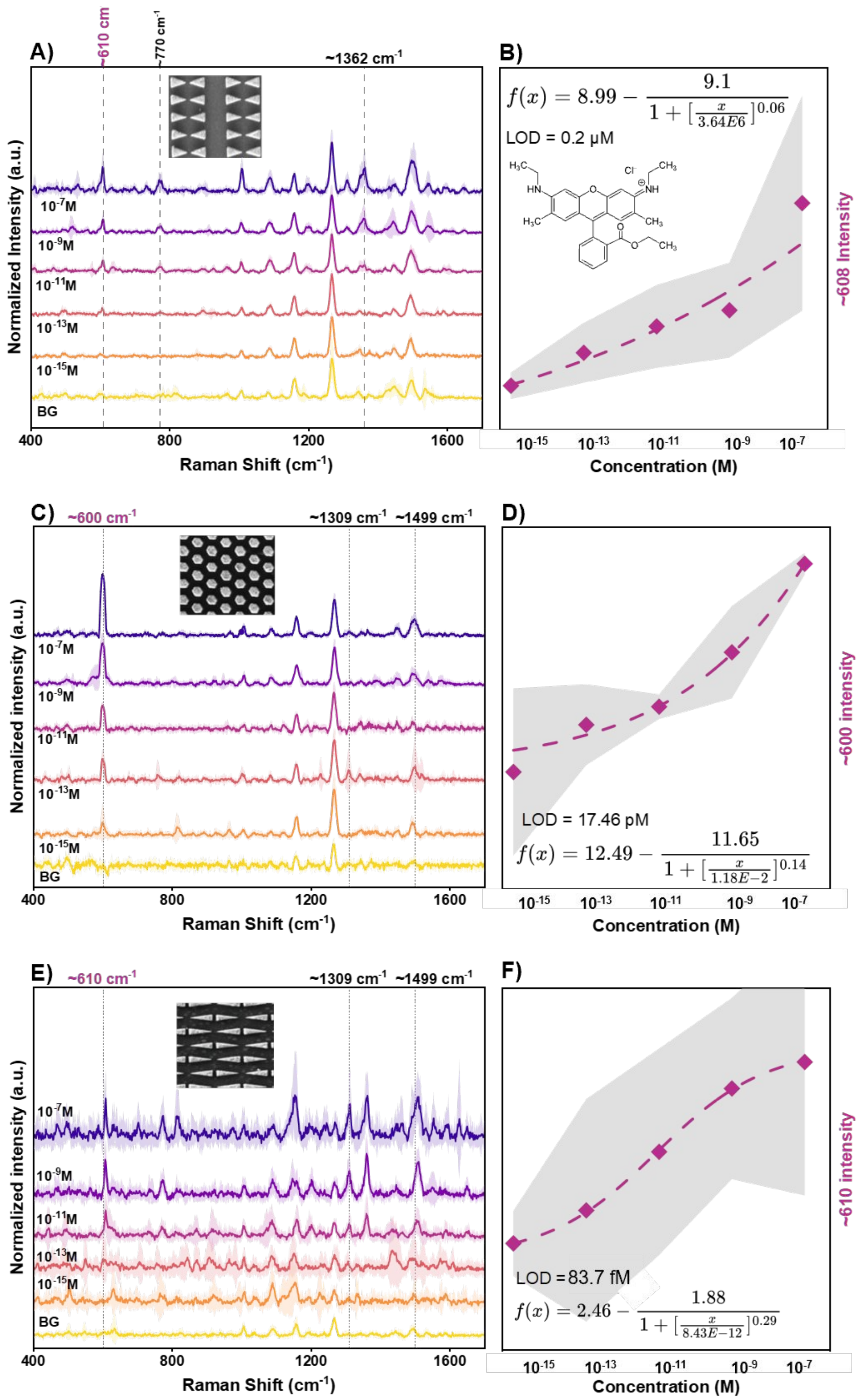

A)
~610 cm
~770 cm-1
~1362 cm-1
Normalized Intensity (a.u.)
10-7M
10-9M
10-11M
10-13M
10-15M
BG
400
800
1200
1600
Raman Shift (cm-1)
B)
$f(x) = 8.99 - \frac{9.1}{1+[\frac{x}{3.64E6}]^{0.06}}$
LOD = 0.2 µM
~608 Intensity
10-15 10-13 10-11 10-9 10-7
Concentration (M)
C)
~600 cm-1
~1309 cm-1 ~1499 cm-1
Normalized intensity (a.u.)
10-7M
10-9M
10-11M
10-13M
10-15M
BG
400
800
1200
1600
Raman Shift (cm-1)
D)
~600 intensity
LOD = 17.46 pM
$f(x) = 12.49 - \frac{11.65}{1+[\frac{x}{1.18E-2}]^{0.14}}$
10-15 10-13 10-11 10-9 10-7
Concentration (M)
E)
~610 cm-1
~1309 cm-1 ~1499 cm-1
Normalized intensity (a.u.)
10-7M
10-9M
10-11M
10-13M
10-15M
BG
400
800
1200
1600
Raman Shift (cm-1)
F)
~610 intensity
LOD = 83.7 fM
$f(x) = 2.46 - \frac{1.88}{1+[\frac{x}{8.43E-12}]^{0.29}}$
10-15 10-13 10-11 10-9 10-7
Concentration (M)

Figure S13. R6G SERS Sensing on the MIM Substrates. A, C & E. Representative spectra obtained on the NTA, HCA, and BTA respectively. B, D & F. R6G Calibration curves on the NTA, HCA, and BTA respectively.

To demonstrate the practicality of SERS detection at the detection limits of the sensors, overlays of background spectra with spectra obtained from analyte concentrations closest to the calculated LOD values are presented for all analytes on the three substrates. The significant prominence of SERS intensity at the representative peak positions with which calibration was constructed in the analyte spectra, relative to the background spectra demonstrates the presence of significant analyte signals at those ultra-low concentrations, despite the noisy backgrounds, showing the practicality of sensing -at the detection limits, of BTA and NTA. Notably, this figure clearly shows the significantly high noise on the HCA substrate, which calls its practicality for univariate sensing close to the calculated detection limit to caution.

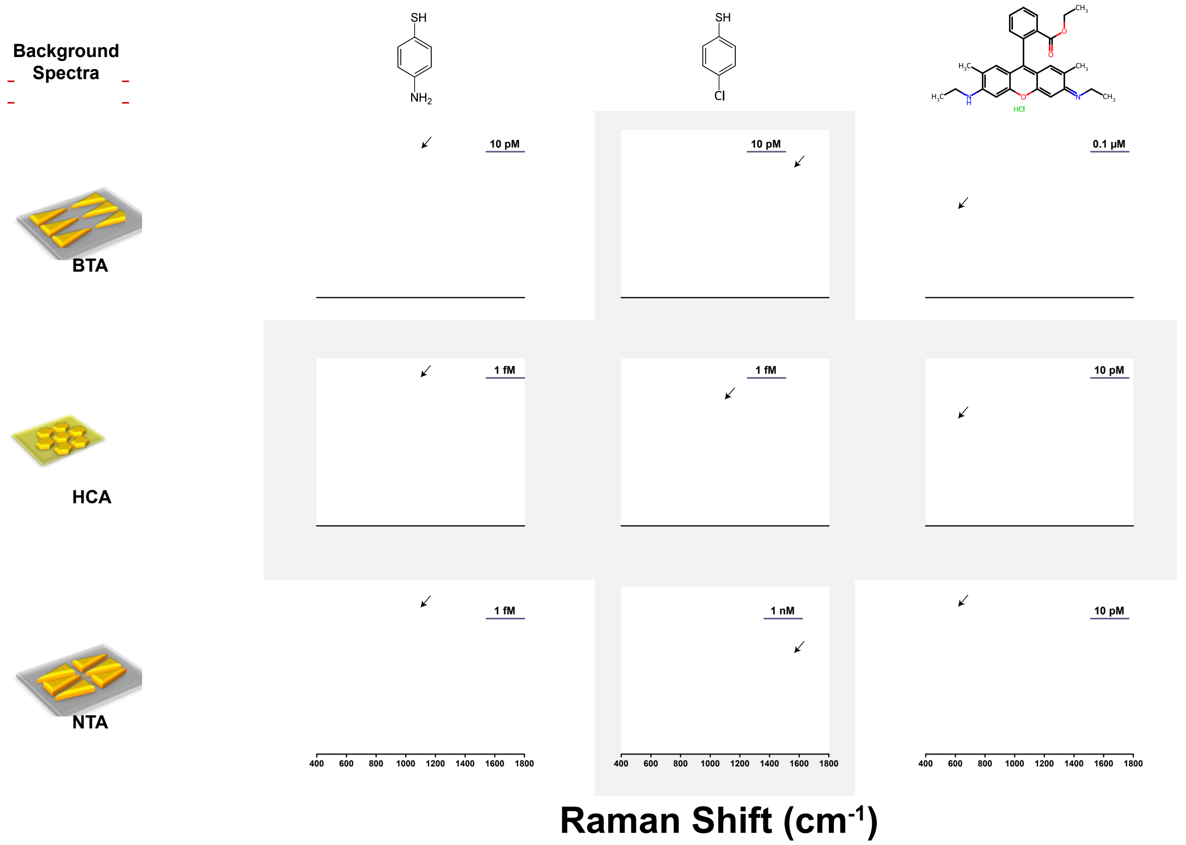

Figure S14. Average background spectra (light pink lines) ± SD (red fills) overlaid with spectra obtained from analyte concentrations closest to the calculated limits of detection (blue) of ATP, CTP, and R6G on the three MIM substrates. The representative SERS peaks with which the logistic calibration curves were constructed are annotated (black arrows).

S13. Comparative SERS Performance of the Fabricated Metasurface Designs

Table S5. Comparative SERS performance of the fabricated metasurface designs (BTA, HCA, and NTA) evaluated using different probe molecules (R6G, 4-ATP, and 4-CTP). Mean Raman intensities, standard deviations, and relative standard deviations (%RSD) were extracted from characteristic vibrational bands at specified Raman shifts over a wide concentration range (10^{-7}–10^{-15} M).

Design	Analyte	Reference Peak	Concentration	Mean Intensity	Mean Stdev	%RSD
BTA	R6G	~610-610 cm^{-1}	1.00E-07	265.16	40.41	15.24
			1.00E-09	238.56	91.23	38.24
			1.00E-11	232.76	67.92	29.18
			1.00E-13	99.11	47.39	47.81
			1.00E-15	90.37	16.90	18.70
HCA	R6G		1.00E-07	151.60	31.59	20.84
			1.00E-09	138.44	32.35	23.37
			1.00E-11	108.94	23.36	21.45
			1.00E-13	117.78	29.49	25.04
			1.00E-15	221.60	70.81	31.95
NTA	R6G		1.00E-07	90.38	26.50	29.32
			1.00E-09	104.16	30.21	29.00
			1.00E-11	138.33	84.06	60.77
			1.00E-13	105.62	82.88	78.47
			1.00E-15	72.85	28.95	39.74
BTA	R6G	~1362 cm^{-1}	1.00E-07	314.31	50.67	16.12
			1.00E-09	193.17	121.15	62.72
			1.00E-11	175.58	103.00	58.66
			1.00E-13	48.92	14.26	29.15
			1.00E-15	88.74	32.67	36.82
HCA	R6G	~1499 cm^{-1}	1.00E-07	318.81	50.13	15.72
			1.00E-09	187.47	38.15	20.35
			1.00E-11	165.96	32.37	19.51
			1.00E-13	146.90	58.14	39.58
			1.00E-15	578.89	322.15	55.65
NTA	R6G		1.00E-07	241.27	66.78	27.68
			1.00E-09	204.61	71.24	34.82
			1.00E-11	195.74	98.97	50.56
			1.00E-13	191.49	75.73	39.55
			1.00E-15	176.02	88.14	50.07
BTA	4-ATP	~1090 cm^{-1}	1.00E-07	1671.63	59.86	3.58
			1.00E-09	718.51	110.74	15.41
			1.00E-11	318.83	47.00	14.74

			1.00E-13	192.64	52.46	27.23
			1.00E-15	67.05	17.16	25.60
**HCA**	4-ATP		1.00E-07	1238.63	189.93	15.33
			1.00E-09	913.43	215.85	23.63
			1.00E-11	746.93	204.36	27.36
			1.00E-13	685.95	256.19	37.35
			1.00E-15	221.09	32.80	14.84
**NTA**	4-ATP		1.00E-07	2454.06	337.12	13.74
			1.00E-09	1388.03	122.97	8.86
			1.00E-11	950.20	212.26	22.34
			1.00E-13	470.32	91.17	19.38
			1.00E-15	219.00	114.09	52.09
**BTA**	4-ATP	~1590 cm^{-1}	1.00E-07	1361.44	137.87	10.13
			1.00E-09	400.41	111.60	27.87
			1.00E-11	208.95	78.08	37.37
			1.00E-13	114.07	20.75	18.19
			1.00E-15	53.25	18.28	34.33
**HCA**	4-ATP		1.00E-07	907.65	207.34	22.84
			1.00E-09	577.32	151.45	26.23
			1.00E-11	414.88	115.53	27.85
			1.00E-13	510.67	191.46	37.49
			1.00E-15	110.31	23.17	21.01
**NTA**	4-ATP		1.00E-07	1253.83	115.30	9.20
			1.00E-09	1015.60	98.81	9.73
			1.00E-11	607.92	169.00	27.80
			1.00E-13	289.13	82.75	28.62
			1.00E-15	135.32	85.62	63.27
**BTA**	4-CTP	~1110 cm^{-1}	1.00E-07	3032.93	485.56	16.01
			1.00E-09	1323.09	133.60	10.10
			1.00E-11	988.70	55.40	5.60
			1.00E-13	780.26	75.27	9.65
			1.00E-15	632.01	133.90	21.19
**HCA**	4-CTP		1.00E-07	1167.81	134.96	11.56
			1.00E-09	342.29	65.71	19.20
			1.00E-11	176.78	37.65	21.30
			1.00E-13	141.28	39.18	27.73
			1.00E-15	76.90	15.45	20.08
**NTA**	4-CTP		1.00E-07	191.88	40.22	20.96
			1.00E-09	73.39	21.15	28.82
			1.00E-11	79.00	25.75	32.59
			1.00E-13	69.83	18.73	26.82
			1.00E-15	57.10	14.71	25.76
**BTA**	4-CTP	~536 cm^{-1}	1.00E-07	3845.94	426.46	11.09

			1.00E-09	1469.39	142.07	9.67
			1.00E-11	856.35	91.44	10.68
			1.00E-13	685.06	79.72	11.64
			1.00E-15	532.55	72.85	13.68
**HCA**	4-CTP		1.00E-07	1967.87	355.98	18.09
			1.00E-09	524.57	65.47	12.48
			1.00E-11	358.28	72.96	20.37
			1.00E-13	286.26	49.89	17.43
			1.00E-15	108.99	29.68	27.23
**NTA**	4-CTP		1.00E-07	289.43	32.80	11.33
			1.00E-09	144.11	33.22	23.05
			1.00E-11	102.10	9.95	9.74
			1.00E-13	82.48	25.12	30.45
			1.00E-15	52.43	15.68	29.90
**BTA**	4-CTP	~1567 cm^{-1}	1.00E-07	6471.99	967.16	14.94
			1.00E-09	1921.64	283.95	14.78
			1.00E-11	1266.77	173.51	13.70
			1.00E-13	707.32	120.38	17.02
			1.00E-15	913.03	176.68	19.35
**HCA**	4-CTP		1.00E-07	1779.99	240.11	13.49
			1.00E-09	559.60	50.32	8.99
			1.00E-11	356.77	66.98	18.77
			1.00E-13	284.90	108.69	38.15
			1.00E-15	141.82	90.25	63.64
**NTA**	4-CTP		1.00E-07	437.46	111.25	25.43
			1.00E-09	153.33	49.50	32.28
			1.00E-11	87.58	25.04	28.59
			1.00E-13	103.06	14.36	13.93
			1.00E-15	79.89	23.80	29.79

S14. Comparative SERS Performance of Different Substrates with MIM

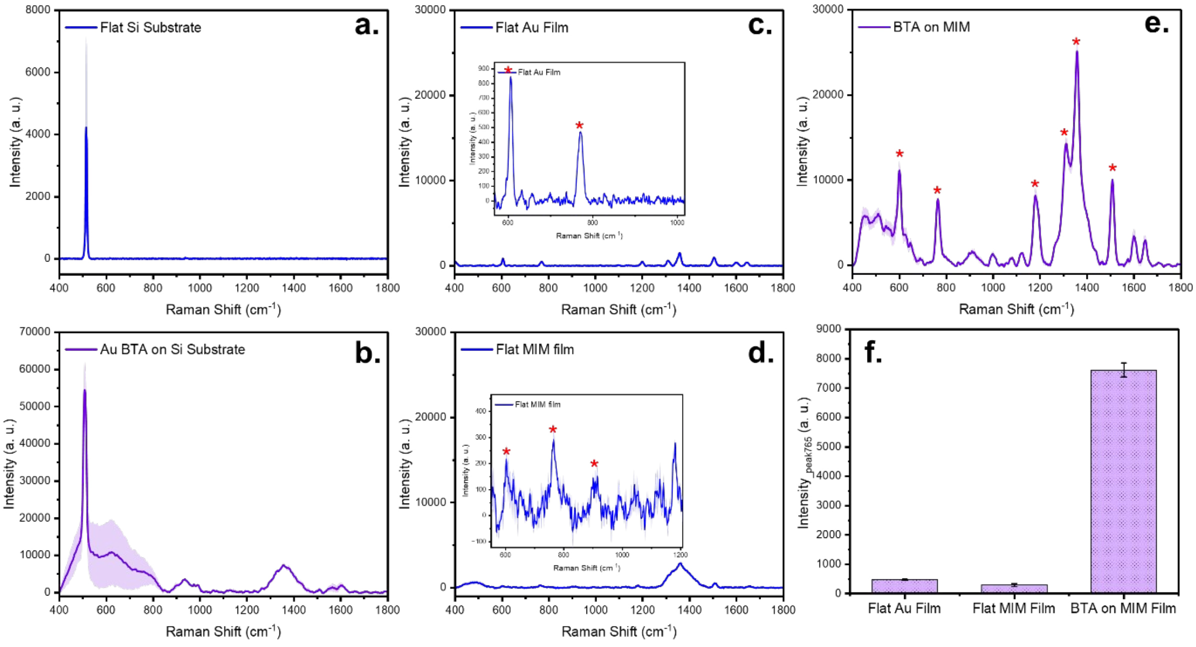

Figure S15. Comparative SERS spectra of 10-4 M R6G measured on bare substrate, flat (unpatterned) MIM, and patterned MIM metasurface structures under identical experimental conditions (60× objective, ~20 uW laser power, wet configuration). All measurements were repeated at least three times ($n \geq 3$). The flat MIM substrate corresponds to the same multilayer architecture fabricated without nanostructures. Characteristic R6G vibrational bands (asterisks) show markedly enhanced intensity on the patterned metasurface. The bar chart presents the quantitative comparison of the peak intensity at ~765 cm-1, demonstrating a significant enhancement for the nanostructured MIM relative to flat MIM and bare substrates (mean ± SD).

S15. Literature Comparison

There have been several simulation and experimental studies in the literature of SERS substrates developed from gold bowtie arrays.[22] However, the Bowtie-MIM-based SERS substrate presented in this study demonstrates significant advantages and differences compared to other approaches in the literature. Firstly, the enhancement factor values of 10^6 obtained both theoretically and experimentally are consistent; this eliminates the problem of insufficient experimental verification despite the theoretical prediction of high EF in many studies and makes our study reliable. Furthermore, the fM limit of detection achieved for 4-ATP demonstrates not only high sensitivity but also practical measurement capabilities. The successful determination of molecules with different functional groups, including R6G, 4-ATP, and 4-CTP, reflects the platform's suitability for a wide range of analyte detection. It is noteworthy that surfaces fabricated by electron beam lithography provide much more regular, reproducible, and controlled nanogap sizes compared to colloidal nanoparticles or self-assembled structures. The MIM architecture used here, unlike traditional bowtie structures, also enables the electromagnetic field to be compressed more intensely in the dielectric layer, allowing more efficient confinement of the optical modes. This, in turn, increases the density of hot spots on the surface, contributing to a stronger and more stable Raman signal. The study also attempts a comparison of the SERS sensing performance of the BTA MIM substrate with that of HCA and NTA substrates.

The bowtie-like NTA structure consists of triangles placed both facing each other and back-to-back, which significantly changes the plasmonic interactions and electromagnetic field localization. An NTA produces a more complex plasmonic response compared to classical single nanoantenna. The back-to-back arrangement of triangles enhances the interaction of plasmonic modes, resulting in a plasmonic hybridization effect and increasing the field intensity over a wider spectral range. As a result, resonance conditions are provided in a wider wavelength range, and Raman signal amplification becomes more effective. An overview of similar MIM designs in literature is presented in **Table S6**.

Table S6. Comparison table of the proposed MIM designs with literature reports.

Structure	Fabrication Method	Detection Molecule	Theoretical EF ($(E/E_0)^4$)	Experimental EF	LOD	Ref.

**Star-disc MIM nanoantenna**	Colloidal lithography	1-octadecanethiol	10^8 (FDTD)	6.58×10^4	10^{-9} M	23
**Au nanosphere MIM**	Colloidal+PECVD	Cyrstal violet and R6G	10^6 (FDTD)	2.62×10^9	10-^{12} M	24
**Au nanosphere MIM**	Colloidal+PECVD	R6G	10^7 (FDTD)	–	10^{-9} M	25
**WO3–PDA/Ag NPs composite**	Colloidal	R6G	-	5.6×10^5	10^{-15} M	26
**Au Bowtie Arrays**	COMSOL Multiphysics	Simulation only	10^8 (FDTD)	–	–	27
**AuNP (rod and sphere)**	Colloidal	R6G	-	10^7	10^{-18} M	28
**Flexible 3D nanofiber**	Nedleless electrospinning	Biphenyl-4-thiol	-	5×10^6	10^{-15} M	29
**Ag NMIM-BNA**	CST Simulation (Notch MIM Bowtie with Ag nanorods)	Simulation only	10^{13}–10^{14} (predicted)	–	–	30

**3D Silver NP Decorated Zinc Oxide/Silicon Heterostructured Nanomace Arrays**	Colloidal lithography	R6G	-	8.7×10^7	10^{-16} M	31
Sliver nanoclusters and PVP micellses	Colloidal	4-ATP	-	10^8	Single molecule	32
**Nanobowtie Microfluidic Device**	Self-assembly (PS monolayer) + lithography (SU8)	Cancer EVs (U373/U87), liposomes	1.1×10^9 (10 nm gap), ~9×10^5 (50–100 nm gap)	3.4×10^5	1.32×10^5 particles/mL	33
**Ag-coated UNGB Arrays**	Holographic lithography + RIE	Benzenethiol	~1×10^8 (simulated, ~5 nm gap)	$5 \times 10^7 – 1 \times 10^8$	–	34
**Au Bowtie TEPL Cavity**	Nanofabricated Au bowtie + movable Au tip	Localized excitons in WSe_2	Purcell effect dominated	≈4.0×10^4	–	35
**Au Bowtie Arrays on PDMS**	EBL + Au evaporation + Ag-assisted transfer to PDMS	MGITC	10^8 (gap shrink from 108 nm →sub-10 nm)	Raman enhancement observed (not numeric EF)	–	36
**Hybrid DHS (Bowtie apertures)**	Optimized hybrid lithography	R6G	500–600 (simulated FE in bowtie gaps)	Raman enhancement vs controls (not absolute EF)	–	37

**Au/Ag Nano-honeycomb on PS Colloidal Beads**	Colloidal lithography	Thiram	-	Not explicitly numeric (enhanced SERS confirmed)	10^{-9} M	38
**PS bCCs-based MIM with Ag shells**	NSL	R6G	10^{4} (between bead to bead)	1.10×10^{8}	10^{-14} M	39
**BTA-HCA-NTA Plasmonic Metasurfaces**	EBL	R6G, 4-ATP, 4-CTP	$<10^{6}$	$<10^{6}$	10^{-15} M	This work

References

(1) Yüce, M.; Öncer, N.; Çınar, C. D.; Günaydın, B. N.; Akçora, Z. İ.; Kurt, H. Comprehensive Raman Fingerprinting and Machine Learning-Based Classification of 14 Pesticides Using a 785 Nm Custom Raman Instrument. *Biosensors (Basel).* **2025**, *15* (3), 168. https://doi.org/10.3390/bios15030168.

(2) López, M. I.; Ruisánchez, I.; Callao, M. P. Figures of Merit of a SERS Method for Sudan I Determination at Traces Levels. *Spectrochim. Acta A Mol. Biomol. Spectrosc.* **2013**, *111*, 237–241. https://doi.org/10.1016/J.SAA.2013.04.031.

(3) Massarini, E.; Wästerby, P.; Landström, L.; Lejon, C.; Beck, O.; Andersson, P. O. Methodologies for Assessment of Limit of Detection and Limit of Identification Using Surface-Enhanced Raman Spectroscopy. *Sens. Actuators B Chem.* **2015**, *207* (Part A), 437–446. https://doi.org/10.1016/J.SNB.2014.09.116.

(4) Seçkin, S.; Singh, P.; Jaiswal, A.; König, T. A. F. Super-Radiant SERS Enhancement by Plasmonic Particle Gratings. *ACS Appl. Mater. Interfaces* **2023**, *15* (36), 43124–43134. https://doi.org/10.1021/acsami.3c07532.

(5) Mathew, E. T.; Serebryannikov, A. E.; Jenczyk, J.; Iatsunskyi, I.; Murawka, S.; Lewandowski, M.; Wiesner, M. Raman Scattering Enhancements Due to Super- and Subradiant Collective Plasmon Modes on Large-Area 2D-Au Arrays. *ACS Appl. Mater. Interfaces* **2025**, *17* (22), 33176–33190. https://doi.org/10.1021/ACSAMI.5C04804.

(6) Seçkin, S.; Singh, P.; Jaiswal, A.; König, T. A. F. Super-Radiant SERS Enhancement by Plasmonic Particle Gratings. *ACS Appl. Mater. Interfaces* **2023**, *15* (36), 43124–43134. https://doi.org/10.1021/acsami.3c07532.

(7) Liu, K. K.; Tadepalli, S.; Tian, L.; Singamaneni, S. Size-Dependent Surface Enhanced Raman Scattering Activity of Plasmonic Nanorattles. *Chemistry of Materials* **2015**, *27* (15), 5261–5270. https://doi.org/10.1021/ACS.CHEMMATER.5B01401.

(8) Hu, X.; Wang, T.; Wang, L.; Dong, S. Surface-Enhanced Raman Scattering of 4-Aminothiophenol Self-Assembled Monolayers in Sandwich Structure with Nanoparticle Shape Dependence: Off-Surface Plasmon Resonance Condition. *Phys. Chem. C* **2007**, *111* (19), 6962–6969. https://doi.org/10.1021/jp0712194.

(9) *Styrene(100-42-5) Raman spectrum*. https://www.chemicalbook.com/SpectrumEN_100-42-5_Raman.htm (accessed 2025-08-28).

(10) *Anisole | C7H8O | CID 7519 - PubChem*. https://pubchem.ncbi.nlm.nih.gov/compound/Anisole#section=Raman-Spectra&fullscreen=true (accessed 2025-08-28).

(11) *Methyl 2-chloroacrylate | C4H5ClO2 | CID 6659 - PubChem*. https://pubchem.ncbi.nlm.nih.gov/compound/Methyl-2-chloroacrylate#section=Raman-Spectra&fullscreen=true (accessed 2025-08-28).

(12) Lee, E. L.; Wachs, I. E. In Situ Raman Spectroscopy of SiO2-Supported Transition Metal Oxide Catalysts: An Isotopic 18O-16O Exchange Study. *Journal of Physical Chemistry C* **2008**, *112* (16), 6487–6498. https://doi.org/10.1021/JP076485W/ASSET/IMAGES/LARGE/JP076485WF00012.JPEG.

(13) Ge, D.; Zhang, Y.; Chen, H.; Zhen, G.; Wang, M.; Jiao, J.; Zhang, L.; Zhu, S. Effect of Patterned Silicon Nitride Substrate on Raman Scattering and Stress of Graphene. *Mater. Des.* **2021**, *198*, 109338. https://doi.org/10.1016/J.MATDES.2020.109338.

(14) Chen, W. L.; Lo, C. Y.; Huang, Y. C.; Wang, Y. C.; Chen, W. H.; Lin, K. J.; Chang, Y. M. Toward Quantitative SERS Detection in Low Analyte Concentration by Investigating the Immersion Volume and Time of SERS Substrate in Analyte Solution. *J. Raman Spectrosc.* **2022**, *53* (1), 33–39. https://doi.org/10.1002/JRS.6250.

(15) Altun, A. O.; Bond, T.; Pronk, W.; Park, H. G. Sensitive Detection of Competitive Molecular Adsorption by Surface-Enhanced Raman Spectroscopy. *Langmuir* **2017**, *33* (28), 6999–7006. https://doi.org/10.1021/ACS.LANGMUIR.7B01186.

(16) Langmuir, I. THE ADSORPTION OF GASES ON PLANE SURFACES OF GLASS, MICA AND PLATINUM. *J. Am. Chem. Soc.* **2002**, *40* (9), 1361–1403. https://doi.org/10.1021/JA02242A004.

(17) Freundlich, H. Über Die Adsorption in Lösungen. *Zeitschrift für Physikalische Chemie* **1907**, *57U* (1), 385–470. https://doi.org/10.1515/ZPCH-1907-5723.

(18) de Oliveira, K. V.; Rubim, J. C. Surface-Enhanced Raman Spectroscopy of Molecules Adsorbed on Silver Nanoparticles Dispersed an Agarose Gel and Their Adsorption Isotherms. *Vib. Spectrosc.* **2016**, *86*, 290–301. https://doi.org/10.1016/J.VIBSPEC.2016.08.006.

(19) Hildebrandt, P.; Stockburger, M. *Surface-Enhanced Resonance Raman Spectroscopy of Rhodamine 6G Adsorbed on Colloidal Silver*; 1984; Vol. 88. https://pubs.acs.org/sharingguidelines.

(20) Jensen, L.; Schatz, G. C. Resonance Raman Scattering of Rhodamine 6G as Calculated Using Time-Dependent Density Functional Theory. *Phys. Chem. A* **2006**, *110* (18), 5973–5977. https://doi.org/10.1021/JP0610867.

(21) Emmons, E. D.; Guicheteau, J. A.; Fountain, A. W.; Tripathi, A. Effect of Substituents on Surface Equilibria of Thiophenols and Isoquinolines on Gold Substrates Studied Using Surface-Enhanced Raman Spectroscopy. *Phys. Chem. Chem. Phys* **2020**, *22* (28), 15953–15965. https://doi.org/10.1039/D0CP01125H.

(22) Arshad, N. S.; Anwar, S.; Wahab, R.; Hussain, A.; Alam, M.; Ali, W.; Awan, T. I.; Nabi, G. Highly Sensitive Plasmonic Au Bowtie Sensors with Extraordinary Optical Absorbance for SERS Applications. *Plasmonics* **2025**, *20* (3), 1427–1442. https://doi.org/10.1007/s11468-024-02388-0.

(23) Li, Y.; Luo, Y.; Lin, X.; Li, D.; Gong, T.; Wang, C.; Yue, W. Metal-Insulator-Metal (MIM) Star-Disc Nanoantenna for Molecular Sensing by Surface-Enhanced Infrared Absorption (SEIRA) Spectroscopy. *Surfaces and Interfaces* **2025**, *57*. https://doi.org/10.1016/j.surfin.2025.105758.

(24) Chen, X.; Cui, A.; He, M.; Yan, M.; Zhang, X.; Ruan, J.; Yang, S. Slippery Au Nanosphere Monolayers with Analyte Enrichment and SERS Enhancement Functions. *Nano Lett.* **2023**, *23* (14), 6736–6743. https://doi.org/10.1021/ACS.NANOLETT.3C02238.

(25) Suganami, Y.; Oshikiri, T.; Mitomo, H.; Sasaki, K.; Liu, Y. E.; Shi, X.; Matsuo, Y.; Ijiro, K.; Misawa, H. Spatially Uniform and Quantitative Surface-Enhanced Raman Scattering under Modal Ultrastrong Coupling Beyond Nanostructure Homogeneity Limits. *ACS Nano* **2024**, *18* (6), 4993–5002. https://doi.org/10.1021/ACSNANO.3C10959.

(26) Tefery, T. G.; Varghese, L.; Jayachitra, R.; Alemu, W. Y.; Nimita Jebaranjitham, J.; Prasannan, A.; Tsai, H. C. Ultra-Sensitive and Enhanced SERS Detection of As3+ and R6G from Self-Assembled WO3–PDA/Ag NPs Composite. *J. Environ. Chem. Eng.* **2025**, *13* (6), 119788. https://doi.org/10.1016/J.JECE.2025.119788.

(27) Venugopalan, P.; Kumar, S. Highly Sensitive Plasmonic Sensor with Au Bow Tie Nanoantennas on SiO2 Nanopillar Arrays. *Chemosensors* **2023**, *11* (2). https://doi.org/10.3390/chemosensors11020121.

(28) Khalil, I.; Yehye, W. A.; Muhd Julkapli, N.; Sina, A. A. I.; Rahmati, S.; Basirun, W. J.; Seyfoddin, A. Dual Platform Based Sandwich Assay Surface-Enhanced Raman Scattering DNA Biosensor for the Sensitive Detection of Food Adulteration. *Analyst* **2020**, *145* (4), 1414–1426. https://doi.org/10.1039/C9AN02106J.

(29) Martino, S.; Yilmaz, D.; Tammaro, C.; Misso, G.; Esposito, A.; Falco, M.; Cossu, A. M.; Lombardi, A.; Amler, E.; Divin, R.; Giannetti, A.; Scrima, M.; Dardano, P.; De Stefano, L.; Rea, I.; De Luca, A. C.; Caraglia, M. Flexible 3D Nanofiber-Based SERS Biosensor for Detection of MiRNA-223-3p in Early Laryngeal Cancer Diagnosis. *Talanta* **2025**, *285*, 127293. https://doi.org/10.1016/J.TALANTA.2024.127293.

(30) Zhang, X.; Guan, R.; Ding, Q.; Wang, C.; Li, Y.; Huang, D.; Chen, Q.; Yang, Z. Exciting High-Order Plasmon Mode Using Metal-Insulator-Metal Bowtie Nanoantenna. *Nanomaterials* **2025**, *15* (12). https://doi.org/10.3390/nano15120882.

(31) Huang, J.; Chen, F.; Zhang, Q.; Zhan, Y.; Ma, D.; Xu, K.; Zhao, Y. 3D Silver Nanoparticles Decorated Zinc Oxide/Silicon Heterostructured Nanomace Arrays as

High-Performance Surface-Enhanced Raman Scattering Substrates. *ACS Appl. Mater. Interfaces* **2015**, *7* (10), 5725–5735. https://doi.org/10.1021/AM507857X.

(32) Cho, W. J.; Kim, Y.; Kim, J. K. Ultrahigh-Density Array of Silver Nanoclusters for SERS Substrate with High Sensitivity and Excellent Reproducibility. *ACS Nano* **2011**, *6* (1), 249–255. https://doi.org/10.1021/NN2035236.

(33) Jalali, M.; Isaac Hosseini, I.; AbdelFatah, T.; Montermini, L.; Wachsmann Hogiu, S.; Rak, J.; Mahshid, S. Plasmonic Nanobowtiefluidic Device for Sensitive Detection of Glioma Extracellular Vesicles by Raman Spectrometry. *Lab Chip* **2021**, *21* (5). https://doi.org/10.1039/d0lc00957a.

(34) Yao, X.; Jiang, S.; Luo, S.; Liu, B. W.; Huang, T. X.; Hu, S.; Zhu, J.; Wang, X.; Ren, B. Uniform Periodic Bowtie SERS Substrate with Narrow Nanogaps Obtained by Monitored Pulsed Electrodeposition. *ACS Appl. Mater. Interfaces* **2020**, *12* (32). https://doi.org/10.1021/acsami.0c09357.

(35) Lee, H.; Kim, I.; Park, C.; Kang, M.; Choi, J.; Jeong, K. Y.; Mun, J.; Kim, Y.; Park, J.; Raschke, M. B.; Park, H. G.; Jeong, M. S.; Rho, J.; Park, K. D. Inducing and Probing Localized Excitons in Atomically Thin Semiconductors via Tip-Enhanced Cavity-Spectroscopy. *Adv. Funct. Mater.* **2021**, *31* (33). https://doi.org/10.1002/adfm.202102893.

(36) Chang, C. Y.; Lin, H. T.; Lai, M. S.; Shieh, T. Y.; Peng, C. C.; Shih, M. H.; Tung, Y. C. Flexible Localized Surface Plasmon Resonance Sensor with Metal–Insulator–Metal Nanodisks on PDMS Substrate. *Sci. Rep.* **2018**, *8* (1). https://doi.org/10.1038/s41598-018-30180-8.

(37) Kabusure, K. M.; Piskunen, P.; Yang, J.; Linko, V.; Hakala, T. K. Raman Enhancement in Bowtie-Shaped Aperture-Particle Hybrid Nanostructures Fabricated with DNA-Assisted Lithography. *Nanoscale* **2023**. https://doi.org/10.1039/d3nr00616f.

(38) Zhang, Y.; Xu, Z.; Wen, J.; Zhao, X.; Gao, R.; Wang, Y. Honeycomb-like Ag Nanocavity Array for SERS Observations Using Plasmon-Mediated Chemical Reactions. *Micromachines (Basel).* **2023**, *14* (10). https://doi.org/10.3390/mi14101811.

(39) Li, W.; Lu, X.; Yang, R.; Liang, F.; Chen, W.; Xie, Z.; Zheng, J.; Zhu, J.; Huang, Y.; Yue, W.; Li, L.; Su, Y. Highly Sensitive and Reproducible SERS Substrates with Binary Colloidal Crystals (BCCs) Based on MIM Structures. *Appl. Surf. Sci.* **2022**, *597*. https://doi.org/10.1016/j.apsusc.2022.153654.
